# Identification and validation of differentially expressed proteins in epithelial ovarian cancers using quantitative proteomics

**DOI:** 10.18632/oncotarget.13077

**Published:** 2016-11-04

**Authors:** Hong Qu, Yuling Chen, Guangming Cao, Chongdong Liu, Jiatong Xu, Haiteng Deng, Zhenyu Zhang

**Affiliations:** ^1^ Department of Obstetrics & Gynecology, Beijing Chao-yang Hospital Affiliated to Capital Medical University, Beijing, China; ^2^ Tsinghua University-Peking University Joint Center for Life Sciences, Beijing, China; ^3^ MOE Key Laboratory of Bioinformatics, School of Life Sciences, Tsinghua University, Beijing, China

**Keywords:** proteomics, epithelial ovarian cancer, CLIC1, LGALS3BP, therapeutic target

## Abstract

Ovarian cancer is the most lethal gynecological malignant tumor because of its high recurrence rate. In the present work, in order to find new therapeutic targets, we identified 8480 proteins in thirteen pairs of ovarian cancer tissues and normal ovary tissues through quantitative proteomics. 498 proteins were found to be differentially expressed in ovarian cancer, which involved in various cellular processes, including metabolism, response to stimulus and biosynthetic process. The expression levels of chloride intracellular channel protein 1 (CLIC1) and lectin galactoside-binding soluble 3 binding protein (LGALS3BP) in epithelial ovarian cancer tissues were significantly higher than those in normal ovary tissues as confirmed by western blotting and immunohistochemistry. The knockdown of CLIC1 in A2780 cell line downregulated expression of CTPS1, leading to the decrease of CTP and an arrest of cell cycle G1 phase, which results into a slower proliferation. CLIC1-knockdown can also slow down the tumor growth in vivo. Besides, CLIC1-knockdown cells showed an increased sensitivity to hydrogen peroxide and cisplatin, suggesting that CLIC1 was involved in regulation of redox and drug resistance in ovarian cancer cells. These results indicate CLIC1 promotes tumorgenesis, and is a potential therapeutic target in epithelial ovarian cancer treatment.

## INTRODUCTION

Ovarian cancer is the fifth leading cause of death and the most lethal gynecological carcinoma in women. It has an annual new cases of 22280 and mortality of 15500, accounting for 3% new cancer cases and 6% of all cancer deaths in women [1]. It is difficult to diagnose epithelial ovarian cancer (EOC) at early stage because of deep location of the organ in pelvis, thus over sixty percent of patients are at advanced stage when first diagnosed [2]. The standard therapy for EOC is surgery followed by platinum–based adjuvant chemotherapy [3]. Although the treatment of EOC is improving continuously, the cure rate of patients at advanced stage still remains less than 5-10% and the 5-year survival rate is less than 30% [4]. Thus there is an urgent need to discover promising therapeutic targets of EOC for improving the outcome.

Over the past decade years, numerous proteomic analysis has been performed to discover molecular targets and proteins associated with the regulation of biological process. In 2002, Petricoin *et al.* [5] firstly employed proteomic patterns with serum of patients to identify ovarian cancer biomarkers, in which the correct identification rate of discriminatory pattern in ovarian cancer could reach above 90%. Subsequently, many proteomic studies aimed to identify biomarker candidates or therapeutic targets in EOC have been carried out. Various clinical samples are used in proteomic analysis, such as serum, tissue, urine and ascites. Wang *et al*. [6] found KRT8 protein, inorganic pyrophosphatase (PPA1), isocitrate dehydrogenase (IDH2), and protein S100-A11 were up-regulated in ovarian cancer tissue using quantitative proteome analysis with an iTRAQ approach. Also, Calcium-Activated Chloride Channel Regulator 1 (CLCA1) was revealed to be overexpressed in ovarian cancer cell [7]. However, due to the limitation of mass spectrometry sensitivity and accuracy in the past, few protein targets can be found in previous studies and used as the potential biomarkers or therapy targets in clinic.

In this study, we identified proteins differentially expressed in ovarian carcinoma compared to normal ovary tissue by using quantitative proteomics analysis. The up-regulation of two proteins, chloride intracellular channel protein 1 (CLIC1) and lectin galactoside-binding soluble 3 binding protein (LGALS3BP), were found in ovarian cancer tissues by quantitative proteomics and confirmed by immunohistochemistry and western blotting. The ovarian cancer cell line A2780 with CLIC1 knock-down showed slower proliferation in vitro and in vivo. CTPS1 (CTP synthase 1) was identified to be down regulated in CLIC1 KD cell line, leading to the decrease of CTP, followed by G1 phase arrest of cell cycle, which results into a slower proliferation rate. In addition, CLIC1-knockdown cells were more sensitive to redox stimulation and cisplatin. From these results, we concluded that CLIC1 promoted tumorgenesis and progression, which is a potential therapeutic target for epithelial ovarian cancer.

## RESULTS

### Quantitative Proteomic Analysis of Ovarian Cancer Tissues and Normal Ovary Tissues

The sample information of patients used in this study was listed in Supplementary Table S1, including 13 epithelial ovarian cancers (6 serous, 3 mucous, 3 clear cell and 1 endometrioid) and 13 normal ovaries with benign gynecologic disease (10 uterine myoma, 1 adenomyosis and 2 with both myoma and adenomyosis). Among the 13 patients with ovarian cancer, 6 were at early stage (46.2%, five at FIGO stage I and one at FIGO stage II), 7 were at advanced stage (53.8%, FIGO stage III). The average age of ovarian cancer group and control group were 54.3 (29-74) and 51.4 (46-56) years, respectively. H&E staining of some tumor samples were showed in Supplementary Figure S1, the average tumor cellularity of all the tumor tissues used was about 75%.

A total of 8480 proteins were identified in all samples with less than 1% FDR. Based on reporter ion ratios (>1.3 or <0.75), 498 proteins were found to be differentially expressed in ovarian cancer tissues with *p*<0.05, in which 369 proteins were significantly down regulated and 129 proteins were up regulated compared with normal ovary tissues (Supplementary Table S2 and S3).

**Figure 1:**
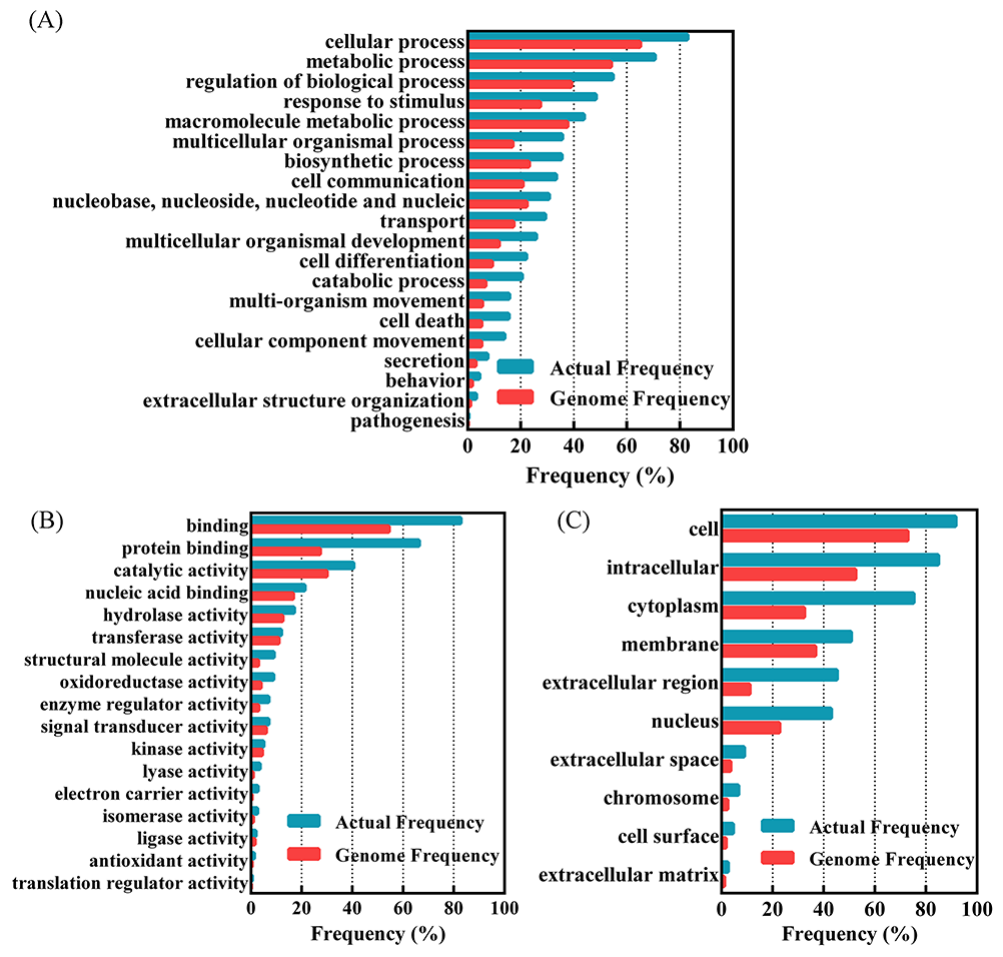
Functional classification of differentially expressed proteins in 3 ovarian cancers compared to normal control with GO Term Finder Mapper (http://go.princeton.edu/cgi-bin/GOTermMapper) **A.** Classified based on protein biological processes; **B.** classification based on the protein functions; **C.** classification based on the protein locations. Genome frequency is the proportion of the genes belonged to a specific classified type in all the human annotated genes, while the actual frequency is the proportion of the genes belonged to a specific classified type in the found proteins with significant difference between ovarian cancers and normal ovarian tissues.

In order to understand the biological function of the identified proteins, the differentially expressed proteins are classified according to protein biological processes, functions and locations. The classification was performed by GO Term Finder Mapper (http://go.princeton.edu/cgi-bin/GOTermMapper) as shown in Figure 1. 498 proteins were significantly enriched in twenty protein biological processes, seventeen protein functions and ten protein locations with *p*<0.05. The enriched groups of protein biological processes including metabolic process, regulation of biological process, response to stimulus, macromolecule metabolic process, biosynthetic process and cell communication, while the enriched groups of protein functions including binding, catalytic activity, transferase and kinase. About 50% of changed proteins are related to membrane or have extracellular regions.

### Verification of overexpressed proteins in ovarian cancer tissues

**Figure 2:**
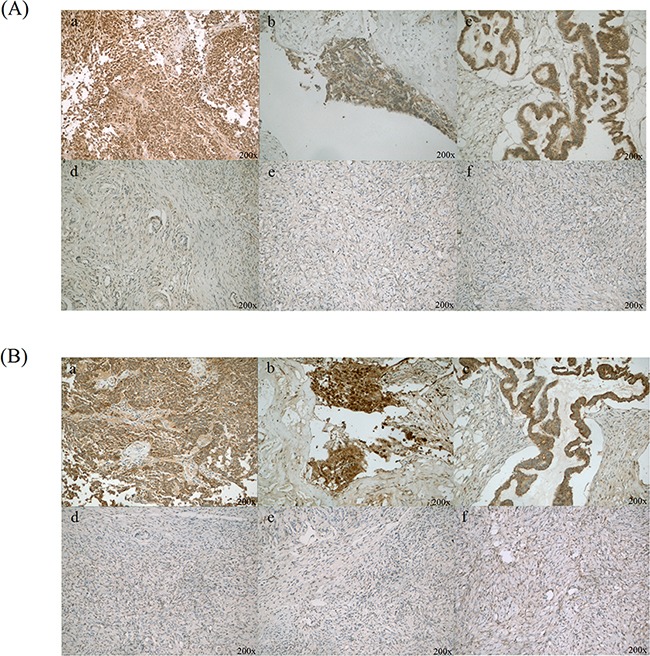
Immunohistochemical staining of CLIC1 and LGALS3BP in ovarian cancer and normal ovary tissues **A.** CLIC1 was positive staining in ovarian cancer cells (a, b and c), as well as benign ovarian epithelial cells were with negative or weak CLIC1 staining (d, e and f); **B.** LGALS3BP was strong positive staining in ovarian cancer cells (a, b and c) and almost negative in normal ovarian epithelial cells (d, e and f). Original magnification: 200×.

In order to find the potential targets of diagnosis and therapy of ovarian cancer, we want to pick up proteins located in cell membrane or secretion of cell for study. Many reports suggest that CLIC1 and LGALS3BP locate in cell membrance and might promote the tumor progression of other cancers. The up regulation of CLIC1 and LGALS3BP in ovarian cancer tissues in quantitative proteomics (Supplementary Figure S2) were verified by western blotting and immunohistochemistry. CLIC1 and LGALS3BP were strongly expressed in 13 ovarian cancer tissues compared with normal ovary tissues by immunohistochemistry. High levels of CLIC1 in ovarian cancer tumor cell cytoplasm and membrane were observed, as well as high levels of LGALS3BP in tumor cell membrane (Figure 2 and Supplementary Figure S3). The positive expression of CLIC1 was 92.3% and 30.8% in ovarian cancer tissues and normal ovary tissues, respectively (*p*<0.001). Also, the positive expression rate of LGALS3BP in ovarian cancer tissues was significant higher than in normal ovary tissues (76.9% vs. 7.7%, *p*<0.001).

**Figure 3:**
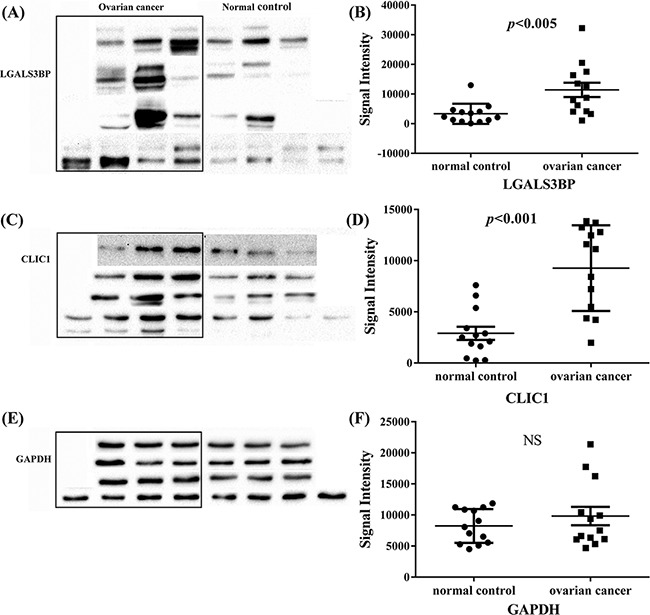
Western blot analysis of LGALS3BP and CLIC1 in all tissue samples of ovarian cancer and normal control **A, C** and **E.** are western blot analysis of LGALS3BP, CLIC1 and GAPDH respectively, GAPDH was used as control; **B, D** and **F.** western blot signal intensities quantitated using Image J 1.48v with the images of these 13 groups of ovarian cancer and normal control. NS indicates no significance.

In addition, the results of western blotting analysis were also shown up regulation of CLIC1 and LGALS3BP (Figure 3A, 3C and 3E). Quantitation of band intensities in western blot images was performed by using the Image J software (Figure 3B, 3D and 3F). The results revealed a statistically significant high level of these two proteins in ovarian cancer tissues compared with normal control (*p*<0.005 and *p*<0.001), indicating CLIC1 or LGALS3BP may accelerate the development of tumor. *CLIC1* and *LGALS3BP* were knocked down by shRAN separately in A2780 cells, but unfortunately the knockdown of *LGALS3BP* in ovarian cancer cells showed no significant phenotypes.

### Establishing of A2780 CLIC1 KD cell line

**Figure 4:**
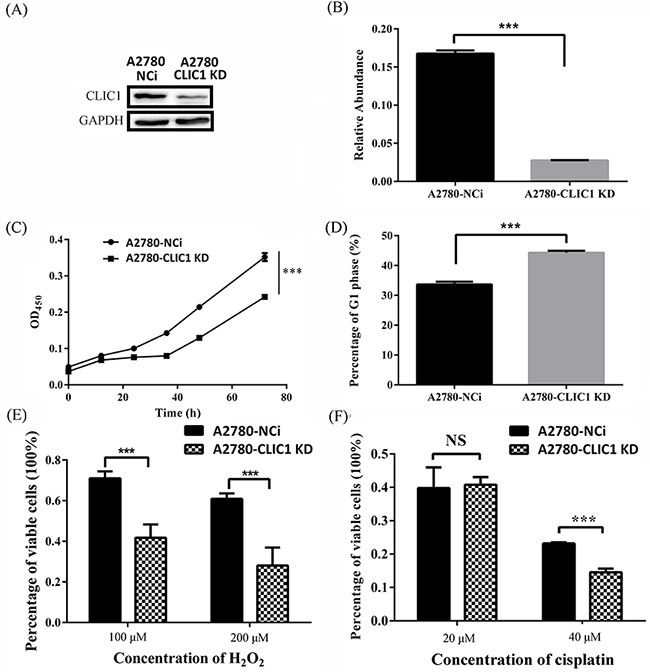
shRNA knockdown of CLIC1 confirms that CLIC1 influence proliferation, resistant to hydrogen peroxide and cisplatin **A.** Western blotting was used to validate the efficiency of CLIC1 knockdown in A2780-CLIC1 KD cells; **B.** q-PCR was used to measure the relative repression ratio of CLIC1 mRNA compared to GAPDH as an internal control in A2780-NCi and A2780-CLIC1 KD cells; **C.** knockdown of CLIC1 in A2780 reduces the growth rate of cells, and cells were observed every 12 h; **D.** the measurement of cell cycle in A2780 NCi and A2780-CLIC1 KD cells; **E.** percentages of variable cells treated with hydrogen peroxide at different concentrations for 24 h determined with CCK-8 assay; **F.** percentages of variable cells treated with cisplatin at different concentrations for 24 h determined with CCK-8 assay. A2780-CLIC1 KD, A2780 cell line with CLIC1 knockdown; A2780-NCi, A2780 cell line with knockdown control shRNA NCi. ***p<0.001; NS, no significance; n=3.

To determine the function of CLIC1 on progression of ovarian cancer, we established A2780 CLIC1 KD cell line by knocking-down the expression of CLIC1 in ovarian cell line A2780. A shRNA against *CLIC1* was inserted into PLL3.7 plasmid with GFP as the reporter gene. The lentiviral particles containing CLIC1 shRNA were transfected into A2780 cells, which were cultured to generate GFP-positive cells. Single GFP-positive cell was sorted by a flow cytometer, and seeded into single well to produce A2780-CLIC1 KD cells. The A2780 cells transfected with shRNA of NCi were used as control. The expression of CLIC1 and the mRNA level of *CLIC1* in A2780-CLIC1 KD and A2780-NCi cells were detected by western blotting and q-PCR respectively (Figure 4A and 4B), showing that the silence of *CLIC1* in A2780 cells was successful.

### Effects of CLIC1 knock down in A2780 cells

Cell proliferation rates of A2780-CLIC1 KD and NCi cells were determined using the CCK-8 assay (Figure 4C). The A2780-CLIC1 KD cells grew more slowly than NCi cells. At 72 h, the number of A2780-CLIC1 KD cells is about 40% less than that of NCi cells. A measurement of cell cycle was also performed, in which a G1 phase arrest of cell cycle was found in A2780-CLIC1 KD cells compared to A2780-NCi cells (Figure 4D). To detect the susceptibility of A2780-CLIC1 KD cells and NCi cells to hydrogen peroxide and cisplatin, cells were treated with different concentrations of hydrogen peroxide or cisplatin respectively for 24 h. Cells viability was measured by the CCK-8 assay. The dose dependent effect of hydrogen peroxide or cisplatin was presented as the viability of cells after 24 h treatment (Figure 4E and 4F). When cells were treated with 100 μM hydrogen peroxide for 24 h, the percentage of viable cells was 70% and 40% for NCi and A2780-CLIC1 KD cells respectively. When the concentration increased to 200μM, the percentage reduced to 60% and 25% for NCi and A2780-CLIC1 KD cells, respectively. Similarly, when the cells were treated with 40 μM cisplatin, the percentage of viable cells was 25% and 15% for NCi and A2780-CLIC1 KD cells, respectively.

### Quantitative proteomics and q-PCR of A2780 CLIC1 KD cells

A quantitative proteomics was performed with A2780-CLIC1 KD cells and A2780-NCi cells to explore the mechanism of CLIC1 during tumorigenesis of ovarian cancer. Total 6297 proteins were found in two independently biological experiments, in which 114 proteins were found to be differently expressed both in these two experiments. 52 proteins were up regulated with ratios >1.3, while 62 proteins were down regulated with ratios <0.75 in A2780-CLIC1 KD cells compared to A2780-NCi cells (Supplementary Table S4 and S5).

**Figure 5:**
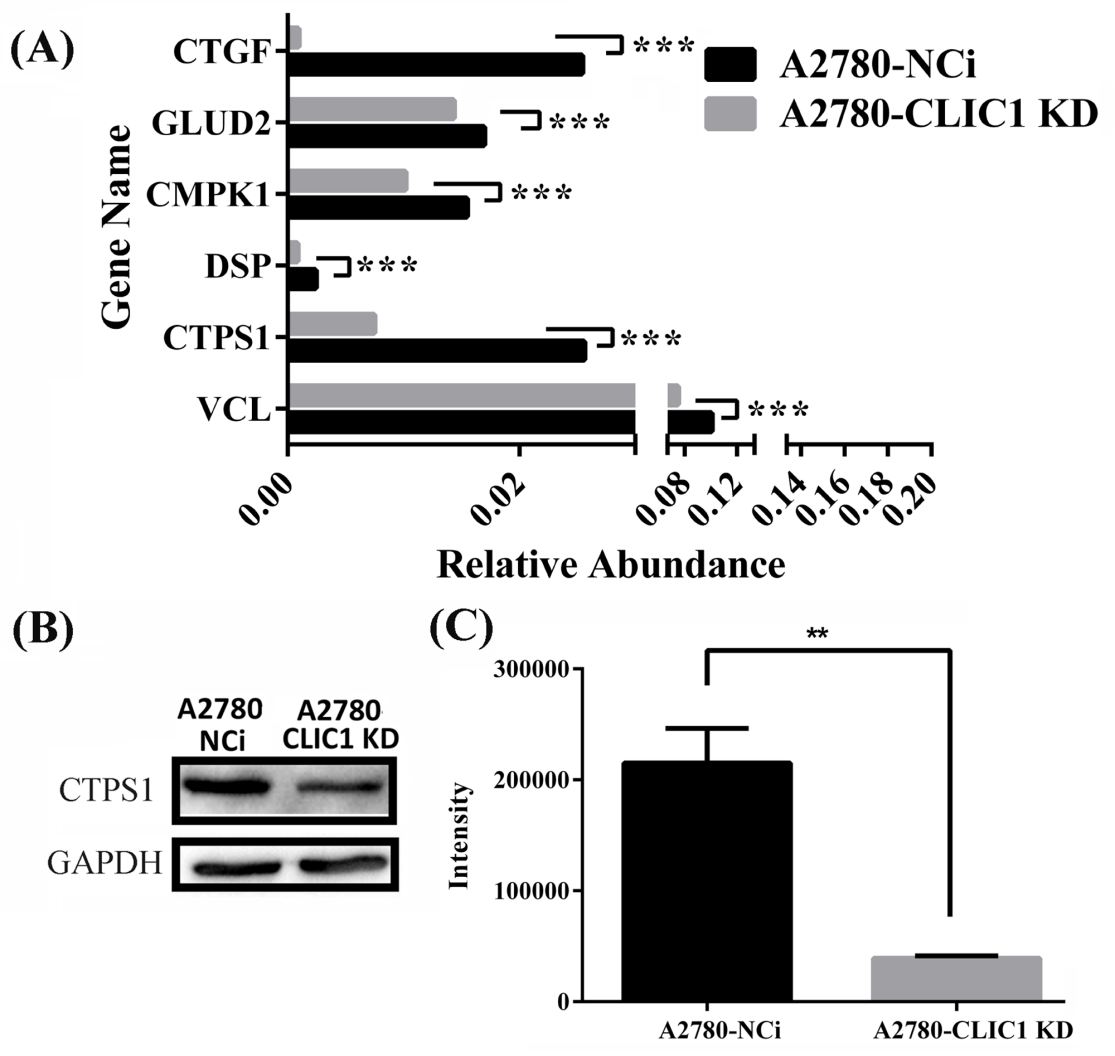
shRNA knockdown of CLIC1 leads to the down regulation of CTPS1, the decrease of CTP concentration and the arrest of G1 phase **A.** q-PCR was used to confirm the down regulation of CTGF, GLUD2, CMPK1, DSP, CTPS1 and VCL on mRNA level; **B.** Western blotting was used to confirm the down regulation of proteins CTPS1; **C.** Metabolomics was used to measure the concentration of CTP in A2780-CLIC1 KD and A2780-NCi cells. A2780-CLIC1 KD, A2780 cell line with CLIC1 knockdown; A2780-NCi, A2780 cell line with knockdown control shRNA NCi. ***p<0.001; **p<0.01; n=3.

To verify the results of quantitative proteomics, the mRNA expression level of genes including connective tissue growth factor (CTGF), glutamate dehydrogenase 2 (GLUD2), UMP-CMP kinase (CMPK1), desmoplakin (DSP), CTP synthase 1 (CTPS1) and isoform 1 of vinculin (VCL) were identified by q-PCR, in which all the mRNA levels were down regulated in A2780-CLIC1 KD cells (Figure 5A). The results of mRNA relative quantification was consistent with the changes of proteomics. The primers used in this study were listed in Supplementary Table S6. The expression of CTPS1 were also verified to be down regulated in A2780-CLIC1 KD cells by western blotting (Figure 5B).

### The measurement of CTP concentration in cells

Metabolomics analysis was carried out to measure the relative concentration of CTP in A2780-CLIC1 KD cells and NCi cells. Metabolites from cells were extracted with a cold mixing solvent which contained 80% methanol and 20% H_2_O. Using TSQ Quantiva™ Triple Quadrupole Mass Spectrometer, relative levels of CTP was determined, showing the concentration of CTP was lower in A2780-CLIC1 KD cells than that in A2780-NCi cells (Figure 5C). The level of CTP in A2780-CLIC1 KD cells was only about a quarter of that in A2780-NCi cells.

### CLIC1 KD cells exhibited decreased in vivo growth rate in nude mice compared to control cells

**Figure 6:**
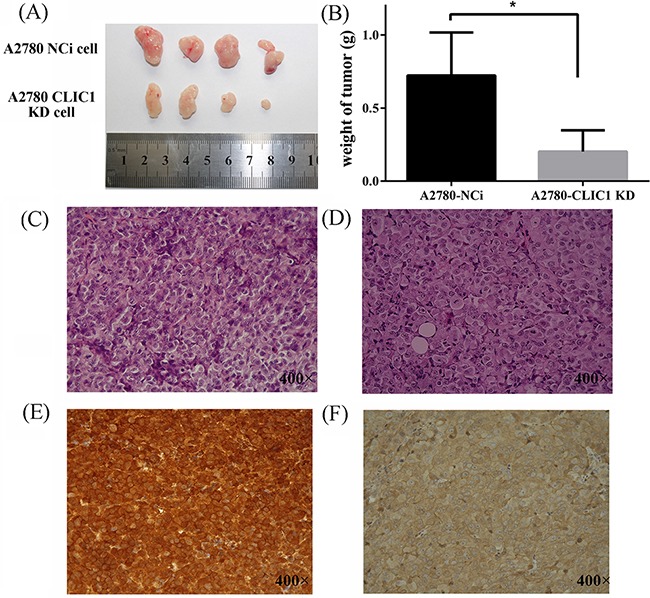
CLIC1 knockdown slows down the tumor growth in vivo in nude mice **A.** Images of tumor samples harvested from mice 28 days after injection of A2780 CLIC1 KD or NCi cells; **B.** The tumors with A2780-CLIC1 KD cells grew much smaller than the tumors with A2780-Nci cells; **C** and **D.** are images of the H&E staining of tissues from mice injected with the A2780-NCi cells and A2780-CLIC1 KD cells respectively; **E** and **F.** are the immunohistochemical staining of CLIC1of tissues from mice injected with the A2780-NCi cells and A2780-CLIC1 KD cells respectively. CLIC1 is strong positive staining in the tissues from mice injected with A2780-Nci cells and almost negative in those with A2780-CLIC1 KD cells. Original magnification: 400×; *p<0.05, n=4.

To test whether CLIC1 accelerates the tumor growth in vivo, injection of A2780-CLIC1 KD cells subcutaneously into 4-week-old immune-compromised mice gave rise to exponentially growing tumors. As shown in Figure 6(A) and 6(B), the tumor with A2780-CLIC1 KD cells grew much smaller than A2780-NCi cells both in volume and weight (*p*<0.05). Then the tumor tissues were identified by H&E staining (Figure 6(C) and 6(D)) and immunohistochemistry of CLIC1 (shown in Figure 6(E) and 6(F)). Xenograft experiment in nude mice demonstrates that CLIC1 knockdown significantly inhibits tumor growth of A2780, providing further evidence to demonstrate that CLIC1 accelerates cancer cell progression.

## DISCUSSION

Target treatment with high sensitivity for ovarian cancer is necessary to increase survival rate of the disease and reduce the side effects. As the development of technology, proteomics provides us a powerful tool to decipher biological processes and more complicated features of proteins including isoforms, modifications and interactions in cancer study [8]. The purpose of this study was to identify and validate proteins that differentially expressed in EOC, which can be potential therapeutic targets in future treatment of EOC.

By quantitative proteomics, 498 proteins out of 8480 identified proteins were differentially expressed between ovarian cancer and normal ovary tissues, in which 129 proteins were up regulated and 369 proteins were down regulated. By GO analysis, the most differentially expressed proteins were related to metabolic process, including carbohydrate metabolism and lipid metabolism. Some proteins located in the cell membrane or secreted out of cell were up regulated in ovarian cancer, such as CLIC1 and LGALS3BP. Notably, the changes of many proteins are different in the four subtypes of ovarian cancer used in quantitative proteomics, for example 5′-nucleotidase is specially higher in endometrial adenocarcinoma sample than the other three subtypes including mucous adenocarcinoma, clear cell carcinoma and serous adenocarcinoma, shown in Supplementary Table S2 and S3.

Lectin galactoside-binding soluble 3 binding protein (LGALS3BP), is a large molecular glycoprotein. High serum or tissue level of LGALS3BP has been confirmed to be associated with various malignant tumor, like breast cancer and metastasis [9]. By using immunohistochemistry and western blotting, we confirmed LGALS3BP was up regulated in ovarian cancer (Figure 2, 3 and Supplementary Figure S2), which was consistent with our quantitative proteomics results (Supplementary Figure S1) and previous reports [10]. The mechanism of LGALS3BP leading to malignant tumor remains uncertain. Fogeron *et al*. [11] reported that LGALS3BP is associated with the centrosome hypertrophy when overexpressed while accumulation of centriolar when reduced expressed. Furthermore, LGALS3BP possibly promotes cancer formation by regulating integrin mediated cell adhesion pathway and angiogenesis through PI3K/AKT pathway [12–14]. But unfortunately, the ovarian cancer cell line with LGALS3BP knockdown shows no significant phenotype in our study.

CLIC1 is firstly found to be overexpressed in activated macrophages and comprises 241 amino acids [15]. As a homologous protein of GST superfamily [16, 17], CLIC1 is involved in the response of oxidative stress by acting as sensor and effector [18]. Under the oxidative and acidic conditions, the monomeric CLIC1 in cytoplasm translocates to membrane and forms oligomer, acting as active anion channel [17, 19, 20]. In recent years, studies show CLIC1 was up regulated mainly in digestive system neoplasm [21], and its high expression is a poor prognosis for diverse tumor patients [21–25]. CLIC1 was found to be transferred by extracellular vesicle and regulation the glioblastoma growth [26]. Besides, CLIC1 possibly regulates the intracellular ROS generation and promotes cancer cell proliferation and migration through ROS/ERK pathway in colon cancer [27]. All of these results suggest that CLIC1 promotes cell malignant transformation and tumor cell proliferation or migration, suggesting it can be a therapeutic target.

In order to explore the biological effect of CLIC1, we constructed A2780 CLIC1 knocked down cell line, and found the proliferation of cells significantly decreased and cell cycle was arrested in G1 phase when *CLIC1* was silenced. A2780-CLIC1 KD cells also show a slower growth rate in vivo in nude mice (Figure 6). These results are agreement with previous studies [27, 28]. CLIC1 was found to be enriched and promote proliferation, colongenic and tumorigenic capacity in the glioblastomas stem cell [29]. A previous report showed that CLIC1 expressed on the plasma membrane of cells only in G2/M phase, and cell cycle would be arrested in G1 if *CLIC1* was silenced, which suggests CLIC1 participated the regulation of cell cycle [30].

Interestingly, we found CTPS1 decreases when CLIC1 was silenced and the concentration of CTP was reduced a lot in A2780-CLIC1 KD cells. CTPS1 is a CTP synthase, which catalyzes CTP biosynthesis from UTP, ATP and glutamine [31, 32]. CTP increases when the cell enters S phase of cell cycle and maintains at a high level [33–39]. The lack of CTP will lead to the cell cycle arrest. Increased CTP concentration was found in cancer cells like primary liver and kidney carcinomas [40]. Loss function of mutation in CTPS1 results in a novel and life-threatening immunodeficiency in human, revealing the central role of CTP in lymphocyte proliferation [41]. CTPS1 has been reported to be regulated by protein kinase A, glycogen synthase kinase 3 or protein kinase C phosphorylation [42]. But few studies report that the expression of CTPS1 is regulated by CLIC1. We found that both of CTPS1 and CLIC1 are up regulated in ovarian cancer, while down regulated in CLIC1 KD cells. CLIC1 may increase the expression of CTPS1, thus further accelerates cell proliferation through the regulation of CTP synthesis. The expression of CTPS1 is decreased as the knockdown of *CLIC1* in cells, which inhibits the synthesis of DNA and RNA, followed by G1 phase arrest of cell cycle.

We also found the A2780-CLIC1 KD cell is susceptible to oxidative stress and cisplatin for that the viability of CLIC1 KD cells was markedly reduced than NCi cells when treated with hydrogen peroxide and cisplatin. These results indicated that CLIC1 protein may protect ovarian cancer cell from injury caused by oxidative stress and chemotherapy drug as an antioxidant enzyme. Oxidative stress and redox imbalance may contribute to the development and progression of cancer [43]. The invasiveness of cancer like prostate cancer could be altered by modulating the extracellular redox state [44], and antioxidant enzymes play an important role in the redox modulation of cancer cells. Based on this study, CLIC1 is expected to be a potential biomarker and therapeutic target in ovarian cancer. However, further studies are needed to elucidate the role of CLIC1 in development and progression in cancer.

Taken together, LGALS3BP and CLIC1 were up regulated in ovarian cancer patients. CTPS1 was downregulated with the knockdown of CLIC1, resulting in the decreased synthesis of CTP, followed by a G1 phase arrest of cell cycle in CLIC1 KD cells. What's more, CLIC1 contributes to the resistant to redox and chemotherapy drugs in cancer cells. Results presented herein suggest CLIC1 may be a potential therapy target in ovarian cancer, contributing to the diagnosis and treatment of ovarian cancer.

## MATERIALS AND METHODS

### Chemicals and reagents

RPMI 1640 medium, phosphate buffer saline (PBS), penicillin/streptomycin and fetal bovine serum (FBS) were purchased from Wisent (Montreal, Canada). Dithiothreitol (DTT), iodoacetamide (IAM), and Polybrene were purchased from Sigma (St Louis, MO). Sequencing grade trypsin was purchased from Promega (Fitchburg, WI). Anti-GAPDH antibody, anti-LGALS3BP antibody and anti-CLIC1 antibody were purchased from Abcam (Cambridge, UK), anti-CTPS1 antibody purchased from Proteintech (Chicago, IL). Anti-mouse and anti-rabbit secondary antibodies were purchased from Cell Signaling Technology (Boston, MA). Cell counting kit-8 was purchased from Dojindo (Kumamoto, Japan). H_2_O_2_ was purchased from Aladdin. PMSF, mass spectrum grade acetonitrile and methanol were purchased from Thermo (Waltham, MA). Protease inhibitor cocktail were purchased from Selleck (Houston, TX).

### Sample preparation

Ovarian tissue samples from 26 patients were obtained from gynecology department of Beijing Chao-yang hospital (Supplementary Table 1). Tissues were collected after approval of Beijing Chao-yang Hospital Ethics Committee and informed consent from patients, and stored immediately in liquid nitrogen until use. None of the ovarian cancer patients received chemotherapy or radiotherapy before surgery.

Tissue samples were grinded in liquid nitrogen and dissolved in PBS containing 8 M urea, 1×protease inhibitor cocktail (Biotool, B14001) and 1 mM PMSF. Samples were then sonicated for 5 min and protein concentrations were measured by the BCA method. Equal amount of proteins from normal ovary tissue samples were taken to pool together and used as the normal control. 100 μg of each samples were reduced with 5 mM DTT and alkylated with 12.5 mM IAM. Samples were diluted with PBS to 1.5 M urea followed by digestion with trypsin of a 1:100 protease/protein ratio at 37°C overnight. The samples were desalted by Sep-Pak columns (Waters, MA). Peptides from different samples were labeled with tandem mass tags (TMT) reagents (Thermo, Pierce Biotechnology) according to the manufacturer's instruction. The TMT labeled peptides were mixed and desalted by Sep-Pak column. For the quantitative proteomics of cell samples, cells were lysed in PBS containing 8 M urea, 1×protease inhibitor cocktail and 1 mM PMSF, and the other procedures were same with tissue samples process.

### Proteomic analysis

The peptides were fractionated by a UPLC3000 system (Dionex, CA) with an XBridgeTM BEH300 C18 column (Waters, MA). Mobile phase A is H_2_O adjusted by ammonium hydroxide to pH 10, and mobile phase B is acetonitrile adjusted by ammonium hydroxide to pH 10. Peptides were separated with the followed gradients: 8% to 18% phase B, 30 min; 18% to 32% phase B, 22 min. 48 fractions were collected, dried by a speedvac, combined into 12 fractions, and re-dissolved in 0.1% formic acid.

For quantitative proteomic analysis, the TMT-labeled peptides were separated by a 120-min gradient elution at a flow rate of 0.250 μl/min with an EASY-nLCII™ integrated nano-HPLC system (Proxeon, Denmark), which is directly interfaced with a Q Exactive mass spectrometer. The analytical column was a fused silica capillary column (75 μm ID, 150 mm length; packed with C-18 resin, Lexington, MA). Mobile phase A consisted of 0.1% formic acid and mobile phase B consisted of 100% acetonitrile and 0.1% formic acid.

The Q Exactive mass spectrometer was operated in the data-dependent acquisition mode using the Xcalibur 2.1.3 software and there was a single full-scan mass spectrum in the Orbitrap (300–1800 m/z, 70000 resolution) with automatic gain control (AGC) target value of 3e6. A data-dependent acquisition method was performed to collect generated MS/MS spectra at 17500 resolution with AGC target of 1e5 and maximum injection time (IT) of 60 ms for top 20 ions observed in each mass spectrum. The isolation window was set at 2 Da width, the dynamic exclusion time was 60 s and the normalized collisional energy (NCE) was set at 30.

### Data analysis

The generated MS/MS spectra were searched against the Uniprot Human database (January 10, 2015; 89105 sequences) using the SEQUEST searching engine in Proteome Discoverer 1.4 software (PD1.4). The search criteria were as follows: full tryptic specificity was required; one missed cleavage was allowed; carbamidomethylation (C) and TMT sixplex (K and N-terminal) were set as the fixed modifications; the oxidation (M) was set as the variable modification; precursor ion mass tolerances were set at 10 ppm for all MS acquired in an Orbitrap mass analyzer; and the fragment ion mass tolerance was set at 20 mmu for all MS2 spectra acquired. The peptide false discovery rate was calculated using Percolator provided by PD1.4. When the q-value was smaller than 1%, the peptide spectrum match was considered to be correct. False discovery was determined based on peptide spectrum match when searched against the reversed decoy database. Peptides only assigned to a given protein group were considered as unique. The false discovery rate was also set to 0.01 for protein identifications. Relative protein quantification was performed using PD1.4 according to manufacturer's instructions on the intensities of six reporter ion per peptide. Quantification was carried out only for proteins with two or more unique peptide matches. Protein ratios were calculated as the median of all peptide hits belonging to a protein. Quantitative precision was expressed as protein ratio variability.

### Western blotting analysis

Tissues were grinded in liquid nitrogen and dissolved in RIPA lysis buffer containing 1 mM cocktail and 1 mM PMSF. Proteins (each 10 μg) were separated by 12% SDS-PAGE gel and transferred onto a PVDF membrane with electro-blotting. After blocking with 5% nonfat milk for 1 h at room temperature, the membrane was incubated overnight at 4°C with primary antibody (1:1000 dilution for anti-LGALS3BP antibody and 1:2000 dilution for anti-CLIC1 antibody), and then incubated with anti-mouse secondary antibody labeled with HRP at room temperature for 1 h. The membrane was detected with ECL reagents (Engreen, China). GAPDH was used as an internal control. For the western blotting of cell samples, cells were lysed in RIPA lysis buffer containing 1 mM cocktail and 1 mM PMSF. The membranes were incubated overnight at 4°C with primary antibody (1: 1000 dilution for anti-CTPS1 antibody, 1:2000 dilution for anti-CLIC1 antibody).

The detected images were quantified using the Image J image processing and analysis software. One-way ANOVA was used to compare the significance among groups of LGALS3BP and CLIC1. GraphPad Prism 6 was used to perform statistical tests. P values <0.05 were considered significant.

### Immunohistochemical analysis

Ovarian cancer tissues and normal ovary tissues were formalin-fixed and paraffin-embedded. After splicing the samples into section, antigen retrieval was performed by autoclaving at 121°C for 15 min in 10 mM sodium citrate buffer (pH 6.0). All samples were washed with PBS for 3 times, and blocked with 2% BSA at 37°C for 10 min. Each sections were incubated with anti-mouse LGALS3BP antibody (1:50 dilution) or anti-mouse CLIC1 antibody (1:75 dilution) overnight at 4°C. Then samples were washed and incubated with anti-mouse secondary antibody with HRP at 37°C for 40 min. Each sample was stained with 3, 3′-diaminobenzine (DAB) in parallel. Expression of CLIC1 and LGALS3BP were evaluated based on the percentage of cell staining (3(≥90%), 2(50–89%), 1(10–49%), or 0 (0–9%)) and the intensity of cell staining (3 (strong), 2 (moderate), 1 (weak), or 0 (no cell staining)). Then the two scores were multiplied by each other and then divided by 3 to get the final score. The final score ≥1 indicated positive staining [45].

### Cell culture

Human ovarian cancer A2780 cell line and human kidney 293T cell line were obtained from the cell bank of the Chinese Academy of Sciences (Shanghai, China). A2780 cells and 293T cells were grown in RPMI 1640 or DMEM medium supplemented with 10% FBS and 1% penicillin/streptomycin at 37°C in a humidified incubator with 5% CO_2_.

### Production of shRNA lentiviral particles

Lentiviral expression vector PLL3.7 with reporter gene of GFP and the package vectors were obtained by courtesy of Dr. Jun Xu (Tongji University, Shanghai, China). The RNA sequence (GGATGAAACCAGTGCTGAA) used to silence *CLIC1* was designed by the GE Healthcare RNAi design tool (http://dharmacon.gelifesciences.com/design-center/). Non-targeting negative control of shRNA (NCi, TTCTCCGAACGTGTCACGT) was also synthesized. The oligonucleotides were annealed and inserted into the PLL3.7 siRNA expression vector to generate shRNA.

Production of lentiviral particles of *CLIC1* shRNA was carried out based on the protocol published by Tiscornia et al. [46]. Briefly, PLL3.7 and packing vectors were co-transfected into 293T cells when the cell reached 80-90% confluence. The cell culture supernatant was collected after 48h and concentrated with PEG6000. Lentiviral particles were resuspended in PBS.

### Isolation of a monoclonal stable cell line with CLIC1 KD and NCi

A2780 cells were cultured in a 6 well plate, and lentiviral particles with 5 μg/mL Polybrene were added into A2780 cells when they reached 40% confluence. After 72 h, a part of cells expressed GFP. Cells were harvested and resuspended in PBS with 2% FBS, then sorted by a flow cytometer with the green fluorescence. A single GFP-positive cell was seeded into a single well of a 96-well plate. The clone with intense and uniform GFP expression was selected and confirmed the efficiency of CLIC1-knocking down by Western Blotting.

### Cell proliferation assay with CCK-8

Cells were seeded in 96-well plated with 2000 cells per well. The proliferation rate was determined by the Cell Counting Kit-8 (CCK-8) according to the manufacturer's protocol (Dojindo Laboratories, Japan). CCK-8 reagent was added into the wells when the cells grew for 0, 12, 24, 36, 48 and 72h. Absorbance at 450 nm was measured after 2h of incubation with CCK-8.

### Susceptibility of cells to hydrogen peroxide and cisplatin

Effects of hydrogen peroxide and cisplatin on cell proliferation of A2780-CLIC1 KD cells and A2780-NCi cells were analyzed with the CCK-8 kit. Cells (5000 each) were seeded into wells of 96-well cell culture microplates and incubated for 24 h before the treatment of hydrogen peroxide and cisplatin. Then cells were treated with hydrogen peroxide (100 and 200 μM) or cisplatin (20 and 40 μM) for 24 h in triplicate. The CCK-8 reagent was added to treated cells and incubated at 37°C for 2h. Absorbance at 450 nm was measured. Cell viability was calculated by the percentage of viable cells with treatment compared to untreated cells. The experiment was repeated 3 times.

### Cell Cycle flow cytometry analysis

1×10^6^ cells were harvested and resuspended in 500 μl of reaction buffer containing Propidium Iodidedye (Solarbio, Beijing). After mixing, cells were incubated for 30 min in the dark. Cell cycle analysis wasperformed on a BD FACSAria III (Becton Dickinson) and analyzed by the CELLQuest™software (Becton Dickinson).

### Quantitative RT-PCR (qPCR)

Total RNA was extracted from cells using Trizol reagent (Invitrogen) and reverse transcribed to cDNA using reverse transcription kit. Quantitative real-time PCR was performed with the Roche LightCycler 480 II Detection System using SYBR green SuperRealPremixs. GAPDH was used as an internal control. Relative expression levels for each reference gene were calculated. The relative expression ratio of a target gene was calculated based on the threshold cycle (Ct) deviation of A2780 CLIC1 KD versus A2780 NCi : Ratio=(2-ΔCt A2780 CLIC1 KD) / (2-ΔCt A2780 NCi)(ΔCt = Ct target-Ct control;). The primers are listed in S6 Table.

### Measurement of CTP concentration

The cells were washed twice with ice-cold PBS, then incubated with 80% methanol (−80°C) for 1 hour at −80°C. The cells were scraped in the extracted buffer on dry ice and washed with more 80% methanol, all the extracted metabolites were combined together and centrifuged at 14000×g for 5 min. The supernatant was collected and the pellet was dissolved in PBS with 8 M urea. BCA assay was used to measure the concentration of protein in pellet solute. The protein concentration was used as the standard to measure equal amount of metabolite, and metabolites were concentrated completely to dryness using a lyophilizer. The dried metabolites were dissolved in 80% methanol and used for LC-MS/MS analysis. To measure the concentration of CTP in A2780-NCi and A2780-CLIC1 KD cells, the TSQ Quantiva™ Triple Quadrupole Mass Spectrometer with positive/negative ion switching was used for targeted quantitation with selective reaction monitoring (SRM). The SRMs were constructed with parameters acquired through optimizing the collision induced fragmentation of purified standards of the given metabolites. The metabolite extracts were passed through a HILIC column that was interfaced with the mass spectrometer. Metabolites were identified based on the retention time on the LC analysis and the accurate mass measured with <5 ppm mass accuracy. Trace Finder was used to identify the peaks and extract the quantitative information.

### Xenograft experiments

All animal studies were approved by the Animal Research Ethics Committee of Beijing Chaoyang Hospital. For xenograft expetiments, 5×10^6^ A2780-CLIC1 KD and A2780-NCi cells were harvested, washed twice with PBS, and resuspended in 200 μL PBS. The cells were injected subcutaneously into the right flank of 4-week-old female nude mice (Vital River Company, China). The mice were examined twice a week for the development of tumors. Tumor samples from the two animal groups were harvested at 28th days after injection to perform pathologic analysis.

## SUPPLEMENTARY FIGURES ADN TABLES










