# Supplementary material for: Identification and validation of differentially expressed proteins in epithelial ovarian cancers using quantitative proteomics
Source: Oncotarget. 2016 Nov 4;7(50):83187–99. doi: 10.18632/oncotarget.13077 (PMC5347761; doi:10.18632/oncotarget.13077)
Supplement: Supplementary file 1 [file oncotarget-07-83187-s001.pdf]

# Identification and validation of differentially expressed proteins in epithelial ovarian cancers using quantitative proteomics

## SUPPLEMENTARY FIGURES ADN TABLES

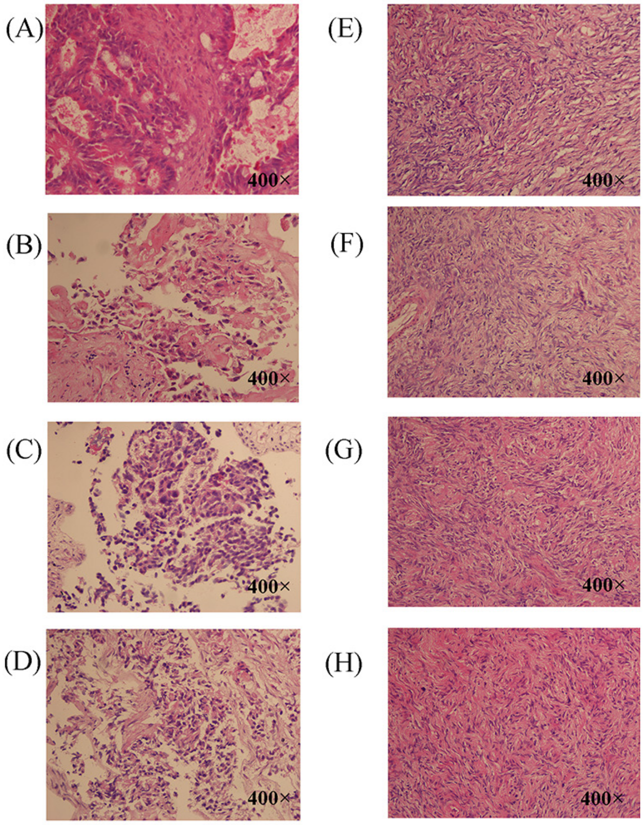


**Supplementary Figure S1: H&E staining of ovarian cancer tissues and normal ovary tissues. A, B, C** and **D.** are images of H&E staining of four ovarian cancer tissue samples; **E, F, G** and **H.** are images of H&E staining of four normal ovary tissue samples. Original magnification: 400×.

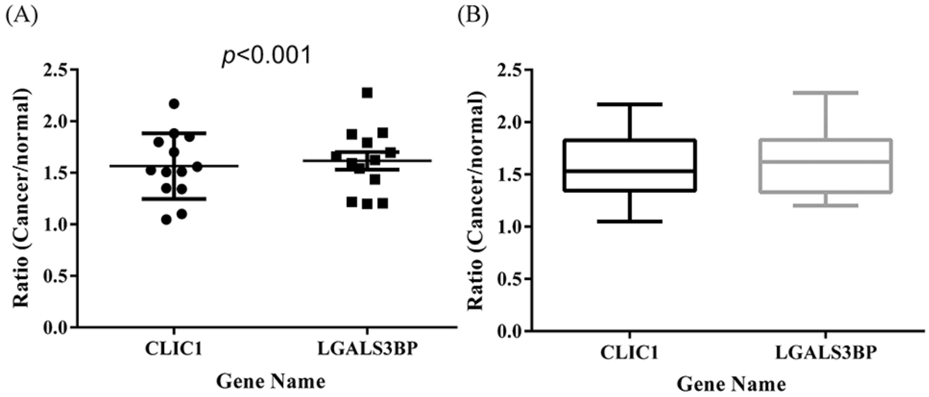


**Supplementary Figure S2: The TMT ratios of CLIC1 and LGALS3BP in 13 ovarian cancer samples compared to normal ovary samples. A.** The scatter diagram of TMT ratios; **B.** the plot box of TMT ratios.

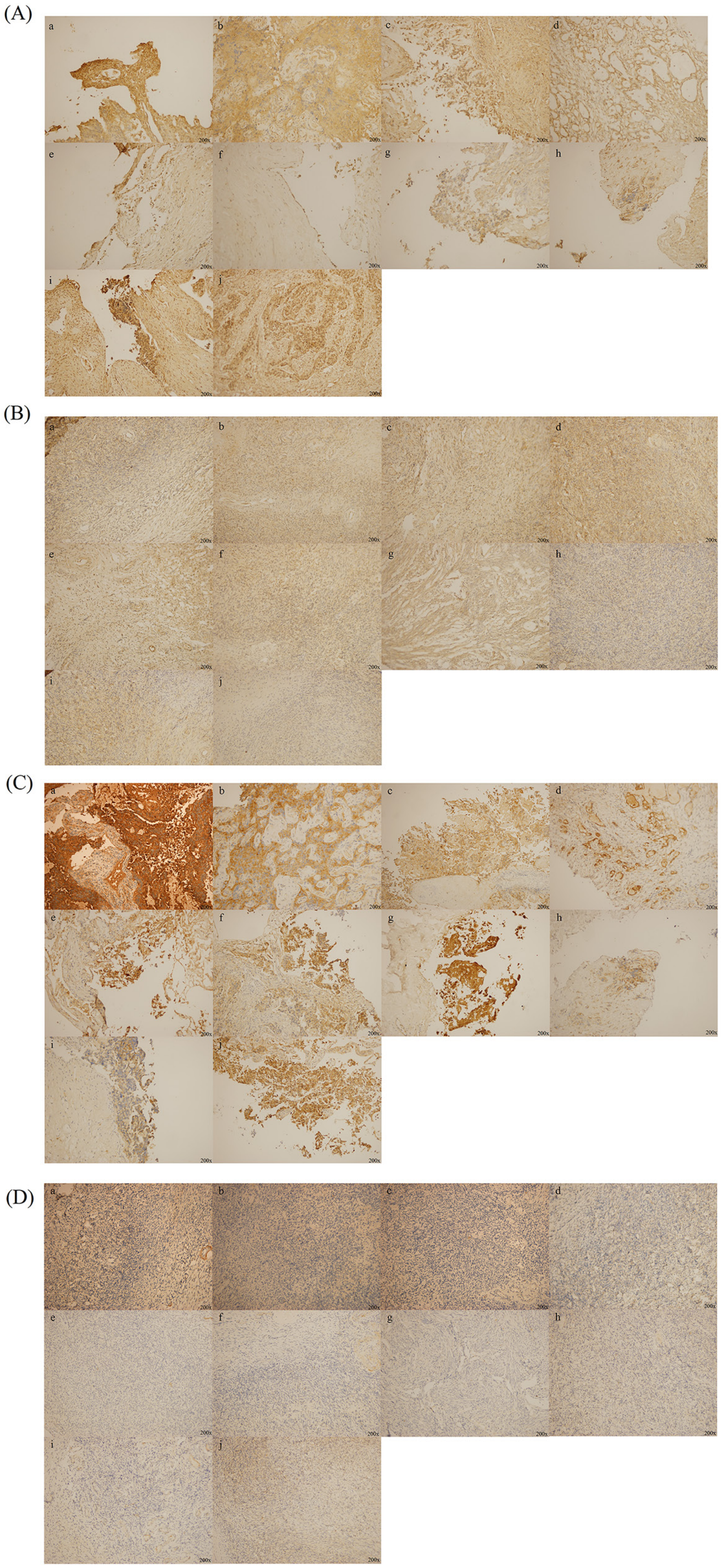


**Supplementary Figure S3: Immunohistochemical staining of CLIC1 and LGALS3BP.** Shown here are epithelial ovarian cancer containing tumor cells with positive staining of CLIC1 **A.** and LGALS3BP **C.**, but normal ovarian epithelial cells are with weak or negative staining of CLIC1 **B.** and LGALS3BP **D.** Original magnification: 200×.

**Supplementary Table S1: The information list of patients for proteomics and western blotting**

| Patients Number | Age | Diagnosis and FIGO Stage |
|---|---|---|
| **OC 1** | 43 | Bilateral ovarian serous adenocarcinoma, stage IIIc |
| **OC 2** | 74 | Right ovarian serous adenocarcinoma, stage IIIc |
| **OC 3** | 61 | Right ovarian serous adenocarcinoma, stage IIIc |
| **OC 4** | 29 | Right ovarian mucinous adenocarcinoma, stage I |
| **OC 5** | 60 | Bilateral ovarian serous papillary adenocarcinoma, stage IIIc |
| **OC 6** | 43 | Bilateral ovarian mucinous adenocarcinoma, stage IIIc |
| **OC 7** | 62 | Left clear cell carcinoma, stage Ic |
| **OC 8** | 44 | Left ovarian clear cell carcinoma, stage Ic |
| **OC 9** | 33 | Right ovarian mucinous adenocarcinoma, stage Ic |
| **OC 10** | 62 | Right ovarian serous adenocarcinoma, stage IIIc |
| **OC 11** | 67 | Right ovarian clear cell carcinoma, stage Ic |
| **OC 12** | 61 | Bilateral ovarian serous adenocarcinoma, stage IIIc |
| **OC 13** | 67 | Left ovarian endometrial adenocarcinoma, IIc |
| **N 1** | 46 | Uterine myoma |
| **N 2** | 49 | Uterine myoma |
| **N 3** | 50 | Uterine myoma |
| **N 4** | 52 | Multiple myoma |
| **N 5** | 56 | Myoma |
| **N 6** | 55 | Multiple myoma, adenomyosis |
| **N 7** | 52 | Myoma |
| **N 8** | 51 | Multiple myoma, adenomyosis |
| **N 9** | 52 | myoma |
| **N 10** | 49 | Myoma |
| **N 11** | 51 | Multiple myoma |
| **N 12** | 53 | adenomyosis |
| **N 13** | 52 | Multiple myoma |

OC stands for the epithelial ovarian cancer patients, while N stands for the patients providing normal ovary tissues.

**Supplementary Table S2: Up regulated proteins in ovarian cancer tissues compared with normal ovarian tissues.**

**See Supplementary File 1**

**Supplementary Table S3: Down regulated proteins in ovarian cancer tissues compared with normal ovarian tissues.**

**See Supplementary File 2**

**Supplementary Table S4: Up regulated proteins in A2780-CLIC1 KD cell line compared with A2780-NCi cell line.**

**See Supplementary File 3**

**Supplementary Table S5: Down regulated proteins in A2780-CLIC1 KD cell line compared withA2780-NCi cell line.**

**See Supplementary File 4**

**Supplementary Table S6: The list of primers used in qPCR**

| Primer Name | Primer Sequence |
|---|---|
| Connective tissue growth factor (CTGF)-F | ACGAGCCCAAGGACCAAA |
| Connective tissue growth factor (CTGF)-R | AGATGCCCATCCCACAGG |
| Glutamate dehydrogenase 2, mitochondrial (GLUD2)-F | GAAAGGGATTCTAACTACCAC |
| Glutamate dehydrogenase 2, mitochondrial (GLUD2)-R | CTCAGATGCACCCGATAT |
| UMP-CMP kinase (CMPK1)-F | CCGCATCGTCGAGAAATA |
| UMP-CMP kinase (CMPK1)-R | TTGGAAACCCATCAATCA |
| Desmoplakin (DSP)-F | GTCACTCAGATGCGAACA |
| Desmoplakin (DSP)-R | CATACTGGACCCTTTGTAAT |
| Chloride intracellular channel protein 1 (CLIC1)-F | TGACAATCTGGAGAAGGGAC |
| Chloride intracellular channel protein 1 (CLIC1)-R | GGACAGGTGGAAGCGAAT |
| CTP synthase 1 (CTPS1)-F | CCTGGGTAACTATGAGCG |
| CTP synthase 1 (CTPS1)-R | TCTGTGATATGAGGGACAAC |
| Isoform 1 of Vinculin(VCL)-F | AGCGAATCCCAACCATAA |
| Isoform 1 of Vinculin(VCL)--R | ACCCAGCGCAGTGTAAAT |
| GAPDH-F | AACGGATTTGGTCGTATTG |
| GAPDH-R | GGAAGATGGTGATGGGATT |
