# Supplementary material for: Identification and validation of differentially expressed proteins in epithelial ovarian cancers using quantitative proteomics
Source: Oncotarget. 2016 Nov 4;7(50):83187–99. doi: 10.18632/oncotarget.13077 (PMC5347761; doi:10.18632/oncotarget.13077)
Supplement: Supplementary file 2 [file oncotarget-07-83187-s002.docx]

Table S2. Up regulated proteins in ovarian cancer tissues compared with normal ovarian tissues.

| Accession | Description | Mean±  SD | *p*-value | Score | Coverage | MW [kDa] | Number of patients (n=13) | proportion in mucinous adenocarci-noma(n=3) | proportion in clear cell carcinoma  (n=3) | proportion in endometrial adenocarci-noma(n=1) | proportion in serous adenocarci-noma(n=6) |
| --- | --- | --- | --- | --- | --- | --- | --- | --- | --- | --- | --- |
| P62280 | 40S ribosomal protein S11 | 1.4±0.2 | 0.00 | 105.4 | 58.9 | 18.4 | 9 | 2/3 | 2/3 | 0/1 | 5/6 |
| P62277 | 40S ribosomal protein S13 | 1.5±0.3 | 0.02 | 144.0 | 56.3 | 17.2 | 8 | 3/3 | 1/3 | 0/1 | 4/6 |
| P62241 | 40S ribosomal protein S8 | 1.5±0.3 | 0.02 | 152.0 | 55.8 | 24.2 | 10 | 3/3 | 3/3 | 0/1 | 4/6 |
| X6RM59 | 5'-nucleotidase | 1.4±0.3 | 0.04 | 34.8 | 22.4 | 37.4 | 8 | 3/3 | 1/3 | 1/1 | 3/6 |
| P40429 | 60S ribosomal protein L13a | 1.4±0.3 | 0.03 | 122.5 | 47.8 | 23.6 | 9 | 2/3 | 3/3 | 0/1 | 4/6 |
| P46778 | 60S ribosomal protein L21 | 1.5±0.4 | 0.04 | 75.7 | 57.5 | 18.6 | 9 | 3/3 | 2/3 | 0/1 | 4/6 |
| P61353 | 60S ribosomal protein L27 | 1.6±0.4 | 0.01 | 33.6 | 46.3 | 15.8 | 10 | 2/3 | 3/3 | 0/1 | 5/6 |
| P46776 | 60S ribosomal protein L27a | 1.5±0.2 | 0.00 | 49.7 | 44.6 | 16.6 | 9 | 2/3 | 2/3 | 1/1 | 4/6 |
| P46779 | 60S ribosomal protein L28 | 1.7±0.4 | 0.01 | 66.5 | 51.1 | 15.7 | 11 | 3/3 | 3/3 | 0/1 | 5/6 |
| P39023 | 60S ribosomal protein L3 | 1.7±0.5 | 0.05 | 163.3 | 48.4 | 46.1 | 11 | 3/3 | 3/3 | 0/1 | 5/6 |
| P62899 | 60S ribosomal protein L31 | 1.4±0.3 | 0.02 | 33.7 | 28.8 | 14.5 | 8 | 2/3 | 3/3 | 0/1 | 3/6 |
| P42766 | 60S ribosomal protein L35 | 2±0.7 | 0.04 | 38.5 | 31.7 | 14.5 | 10 | 2/3 | 3/3 | 0/1 | 5/6 |
| P18077 | 60S ribosomal protein L35a | 1.4±0.2 | 0.01 | 59.9 | 55.5 | 12.5 | 8 | 2/3 | 3/3 | 0/1 | 3/6 |
| P36578 | 60S ribosomal protein L4 | 1.6±0.5 | 0.05 | 213.9 | 51.3 | 47.7 | 9 | 2/3 | 3/3 | 0/1 | 4/6 |
| P62424 | 60S ribosomal protein L7a | 1.7±0.3 | 0.00 | 185.6 | 57.5 | 30.0 | 12 | 3/3 | 3/3 | 0/1 | 6/6 |
| P62917 | 60S ribosomal protein L8 | 1.6±0.4 | 0.01 | 92.3 | 50.6 | 28.0 | 11 | 3/3 | 3/3 | 0/1 | 5/6 |
| O15143 | Actin-related protein 2/3 complex subunit 1B | 1.5±0.3 | 0.00 | 210.4 | 58.6 | 40.9 | 11 | 3/3 | 3/3 | 0/1 | 5/6 |
| O15145 | Actin-related protein 2/3 complex subunit 3 | 1.4±0.2 | 0.01 | 79.4 | 63.5 | 20.5 | 8 | 2/3 | 2/3 | 0/1 | 4/6 |
| P59998 | Actin-related protein 2/3 complex subunit 4 | 1.4±0.2 | 0.01 | 68.6 | 48.8 | 19.7 | 7 | 1/3 | 2/3 | 0/1 | 4/6 |
| A0A087X1K9 | Acyl-protein thioesterase 1 | 1.5±0.3 | 0.05 | 70.3 | 68.1 | 18.0 | 7 | 1/3 | 2/3 | 1/1 | 3/6 |
| P27144 | Adenylate kinase 4, mitochondrial | 1.6±0.5 | 0.05 | 71.5 | 67.3 | 25.3 | 7 | 1/3 | 1/3 | 1/1 | 4/6 |
| P05141 | ADP/ATP translocase 2 | 1.5±0.3 | 0.01 | 453.4 | 60.1 | 32.8 | 9 | 2/3 | 2/3 | 1/1 | 4/6 |
| Q9BYC5 | Alpha-(1,6)-fucosyltransferase | 1.5±0.3 | 0.01 | 55.4 | 32.9 | 66.5 | 7 | 2/3 | 0/3 | 1/1 | 4/6 |
| P00966 | Argininosuccinate synthase | 1.6±0.4 | 0.01 | 238.6 | 63.1 | 46.5 | 11 | 2/3 | 2/3 | 1/1 | 6/6 |
| P15289 | Arylsulfatase A | 1.4±0.3 | 0.02 | 25.0 | 5.9 | 53.6 | 8 | 1/3 | 2/3 | 1/1 | 4/6 |
| P03928 | ATP synthase protein 8 | 1.4±0.3 | 0.03 | 16.4 | 55.9 | 8.0 | 7 | 3/3 | 1/3 | 0/1 | 3/6 |
| P28288 | ATP-binding cassette sub-family D member 3 | 1.5±0.4 | 0.04 | 49.9 | 23.1 | 75.4 | 10 | 3/3 | 3/3 | 1/1 | 3/6 |
| Q9Y6X5 | Bis(5'-adenosyl)-triphosphatase ENPP4 | 1.6±0.4 | 0.01 | 11.5 | 7.5 | 51.6 | 10 | 2/3 | 2/3 | 1/1 | 5/6 |
| Q9UHR4 | Brain-specific angiogenesis inhibitor 1-associated protein 2-like protein 1 | 1.8±0.3 | 0.00 | 18.9 | 13.9 | 56.8 | 11 | 3/3 | 3/3 | 1/1 | 4/6 |
| Q9UJS0 | Calcium-binding mitochondrial carrier protein Aralar2 | 1.8±0.5 | 0.01 | 278.2 | 53.9 | 74.1 | 11 | 3/3 | 3/3 | 1/1 | 4/6 |
| O75976 | Carboxypeptidase D | 1.3±0.2 | 0.05 | 16.9 | 2.5 | 152.8 | 7 | 1/3 | 2/3 | 0/1 | 4/6 |
| P50416 | Carnitine O-palmitoyltransferase 1, liver isoform | 1.4±0.3 | 0.01 | 108.2 | 29.2 | 88.3 | 7 | 2/3 | 1/3 | 0/1 | 4/6 |
| P42574 | Caspase-3 | 1.7±0.3 | 0.00 | 16.3 | 15.5 | 31.6 | 12 | 3/3 | 3/3 | 1/1 | 5/6 |
| Q5SVL2 | Caspase-7 (Fragment) | 1.8±0.4 | 0.05 | 11.5 | 26.1 | 24.8 | 8 | 1/3 | 1/3 | 1/1 | 5/6 |
| Q9Y5K6 | CD2-associated protein | 1.4±0.3 | 0.03 | 91.3 | 32.4 | 71.4 | 7 | 2/3 | 1/3 | 0/1 | 4/6 |
| O75503 | Ceroid-lipofuscinosis neuronal protein 5 | 1.4±0.3 | 0.03 | 15.1 | 11.5 | 41.5 | 8 | 2/3 | 2/3 | 1/1 | 3/6 |
| O00299 | Chloride intracellular channel protein 1 | 1.6±0.3 | 0.01 | 228.5 | 72.2 | 26.9 | 11 | 3/3 | 3/3 | 0/1 | 5/6 |
| Q14019 | Coactosin-like protein | 1.5±0.3 | 0.02 | 99.4 | 93.0 | 15.9 | 9 | 3/3 | 2/3 | 1/1 | 3/6 |
| Q9Y678 | Coatomer subunit gamma-1 | 1.4±0.1 | 0.00 | 330.0 | 54.8 | 97.7 | 9 | 1/3 | 3/3 | 1/1 | 4/6 |
| P61923 | Coatomer subunit zeta-1 | 1.4±0.2 | 0.01 | 57.3 | 27.1 | 20.2 | 7 | 1/3 | 2/3 | 1/1 | 3/6 |
| B7Z2Y2 | Conserved oligomeric Golgi complex subunit 2 | 1.4±0.2 | 0.02 | 15.5 | 9.3 | 76.0 | 7 | 2/3 | 0/3 | 1/1 | 4/6 |
| Q9P1F3 | Costars family protein ABRACL | 2.7±1.3 | 0.05 | 16.0 | 49.4 | 9.1 | 11 | 3/3 | 2/3 | 1/1 | 5/6 |
| P17812 | CTP synthase 1 | 1.5±0.3 | 0.03 | 70.3 | 22.8 | 66.6 | 7 | 3/3 | 1/3 | 0/1 | 3/6 |
| Q96HD1 | Cysteine-rich with EGF-like domain protein 1 | 1.5±0.3 | 0.01 | 30.9 | 16.7 | 45.4 | 9 | 2/3 | 2/3 | 1/1 | 4/6 |
| P04839 | Cytochrome b-245 heavy chain | 1.9±0.6 | 0.02 | 81.5 | 14.4 | 65.3 | 11 | 3/3 | 3/3 | 0/1 | 5/6 |
| P54886 | Delta-1-pyrroline-5-carboxylate synthase | 1.7±0.4 | 0.01 | 273.3 | 50.7 | 87.2 | 10 | 1/3 | 3/3 | 1/1 | 5/6 |
| Q01459 | Di-N-acetylchitobiase | 1.5±0.3 | 0.01 | 18.5 | 15.6 | 43.7 | 10 | 2/3 | 2/3 | 1/1 | 5/6 |
| P53634 | Dipeptidyl peptidase 1 | 1.5±0.3 | 0.02 | 71.2 | 25.3 | 51.8 | 9 | 2/3 | 2/3 | 1/1 | 4/6 |
| Q9NY33 | Dipeptidyl peptidase 3 | 1.5±0.3 | 0.02 | 175.8 | 39.6 | 82.5 | 10 | 2/3 | 2/3 | 1/1 | 5/6 |
| Q13217 | DnaJ homolog subfamily C member 3 | 1.4±0.2 | 0.00 | 82.4 | 45.4 | 57.5 | 10 | 2/3 | 3/3 | 1/1 | 4/6 |
| Q8TDB6 | E3 ubiquitin-protein ligase DTX3L | 1.5±0.3 | 0.01 | 140.7 | 46.8 | 83.5 | 9 | 2/3 | 2/3 | 0/1 | 5/6 |
| Q96C19 | EF-hand domain-containing protein D2 | 1.4±0.2 | 0.01 | 54.1 | 52.9 | 26.7 | 7 | 2/3 | 2/3 | 1/1 | 2/6 |
| Q9UKM7 | Endoplasmic reticulum mannosyl-oligosaccharide 1,2-alpha-mannosidase | 1.4±0.2 | 0.00 | 14.0 | 6.3 | 79.5 | 8 | 2/3 | 3/3 | 1/1 | 2/6 |
| Q92817 | Envoplakin | 1.4±0.2 | 0.02 | 110.6 | 19.4 | 231.5 | 8 | 2/3 | 2/3 | 1/1 | 3/6 |
| Q96HE7 | ERO1-like protein alpha | 1.6±0.4 | 0.03 | 98.0 | 45.9 | 54.4 | 9 | 3/3 | 3/3 | 0/1 | 3/6 |
| E7EW84 | Exocyst complex component 6 | 1.5±0.3 | 0.01 | 13.3 | 7.6 | 81.8 | 10 | 3/3 | 3/3 | 1/1 | 3/6 |
| Q53EP0 | Fibronectin type III domain-containing protein 3B | 1.4±0.2 | 0.00 | 72.1 | 17.7 | 132.8 | 8 | 1/3 | 2/3 | 1/1 | 4/6 |
| Q12841 | Follistatin-related protein 1 | 1.4±0.3 | 0.04 | 8.6 | 9.4 | 35.0 | 8 | 3/3 | 2/3 | 1/1 | 2/6 |
| Q9NQ88 | Fructose-2,6-bisphosphatase TIGAR | 1.7±0.3 | 0.00 | 17.9 | 19.6 | 30.0 | 12 | 3/3 | 3/3 | 1/1 | 5/6 |
| Q08380 | Galectin-3-binding protein | 1.6±0.3 | 0.00 | 196.6 | 34.7 | 65.3 | 10 | 2/3 | 3/3 | 1/1 | 4/6 |
| O60547 | GDP-mannose 4,6 dehydratase | 1.4±0.2 | 0.01 | 83.7 | 49.7 | 41.9 | 8 | 3/3 | 2/3 | 1/1 | 2/6 |
| Q6PCE3 | Glucose 1,6-bisphosphate synthase | 1.4±0.2 | 0.01 | 11.4 | 6.9 | 70.4 | 7 | 2/3 | 1/3 | 0/1 | 4/6 |
| O76003 | Glutaredoxin-3 | 1.4±0.3 | 0.02 | 42.6 | 29.6 | 37.4 | 8 | 1/3 | 3/3 | 1/1 | 3/6 |
| Q9Y2Q3 | Glutathione S-transferase kappa 1 | 1.6±0.4 | 0.03 | 232.7 | 67.7 | 25.5 | 8 | 2/3 | 2/3 | 1/1 | 3/6 |
| Q70UQ0 | Inhibitor of nuclear factor kappa-B kinase-interacting protein | 1.5±0.4 | 0.05 | 62.7 | 35.1 | 39.3 | 7 | 2/3 | 1/3 | 0/1 | 4/6 |
| Q15181 | Inorganic pyrophosphatase | 1.8±0.5 | 0.03 | 241.5 | 87.9 | 32.6 | 11 | 2/3 | 2/3 | 1/1 | 6/6 |
| Q9NX62 | Inositol monophosphatase 3 | 1.6±0.4 | 0.02 | 23.8 | 22.0 | 38.7 | 8 | 2/3 | 2/3 | 0/1 | 4/6 |
| P46734-2 | Isoform 1 of Dual specificity mitogen-activated protein kinase kinase 3 | 1.4±0.3 | 0.03 | 51.3 | 28.3 | 36.1 | 8 | 2/3 | 3/3 | 1/1 | 2/6 |
| Q9BT22-2 | Isoform 2 of Chitobiosyldiphosphodolichol beta-mannosyltransferase | 1.3±0.2 | 0.04 | 17.2 | 13.0 | 40.3 | 7 | 1/3 | 1/3 | 1/1 | 4/6 |
| Q8NBJ4-2 | Isoform 2 of Golgi membrane protein 1 | 1.3±0.2 | 0.04 | 16.0 | 15.9 | 44.2 | 7 | 2/3 | 2/3 | 1/1 | 2/6 |
| P10619-2 | Isoform 2 of Lysosomal protective protein | 1.6±0.3 | 0.00 | 69.9 | 24.0 | 52.5 | 9 | 2/3 | 3/3 | 1/1 | 3/6 |
| Q9NYL4-2 | Isoform 2 of Peptidyl-prolyl cis-trans isomerase FKBP11 | 1.6±0.3 | 0.01 | 56.6 | 33.6 | 15.8 | 9 | 3/3 | 2/3 | 0/1 | 4/6 |
| Q8IXQ6-2 | Isoform 2 of Poly [ADP-ribose] polymerase 9 | 1.4±0.2 | 0.01 | 147.7 | 39.6 | 92.2 | 8 | 2/3 | 2/3 | 0/1 | 4/6 |
| O43665-2 | Isoform 2 of Regulator of G-protein signaling 10 | 1.5±0.3 | 0.04 | 32.1 | 34.1 | 19.6 | 9 | 1/3 | 3/3 | 1/1 | 4/6 |
| P52630-4 | Isoform 2 of Signal transducer and activator of transcription 2 | 1.4±0.2 | 0.00 | 53.2 | 11.9 | 97.4 | 8 | 2/3 | 2/3 | 0/1 | 4/6 |
| Q6RW13-2 | Isoform 2 of Type-1 angiotensin II receptor-associated protein | 1.6±0.4 | 0.02 | 10.4 | 14.5 | 16.7 | 9 | 1/3 | 3/3 | 1/1 | 4/6 |
| Q6FI81-3 | Isoform 3 of Anamorsin | 1.5±0.3 | 0.00 | 22.8 | 26.4 | 32.2 | 8 | 2/3 | 2/3 | 1/1 | 3/6 |
| Q0VD83-3 | Isoform 3 of Apolipoprotein B receptor | 1.6±0.3 | 0.03 | 5.9 | 1.9 | 113.0 | 9 | 3/3 | 2/3 | 0/1 | 4/6 |
| Q6NXG1-3 | Isoform 3 of Epithelial splicing regulatory protein 1 | 1.5±0.3 | 0.03 | 48.5 | 25.6 | 75.1 | 8 | 0/3 | 3/3 | 1/1 | 4/6 |
| Q99538-3 | Isoform 3 of Legumain | 1.4±0.3 | 0.04 | 88.2 | 17.2 | 42.0 | 7 | 2/3 | 3/3 | 0/1 | 2/6 |
| P08631-3 | Isoform 3 of Tyrosine-protein kinase HCK | 1.5±0.3 | 0.02 | 31.0 | 19.1 | 57.2 | 7 | 2/3 | 3/3 | 0/1 | 2/6 |
| Q70UQ0-4 | Isoform 4 of Inhibitor of nuclear factor kappa-B kinase-interacting protein | 1.4±0.3 | 0.04 | 94.1 | 47.2 | 43.1 | 9 | 2/3 | 2/3 | 0/1 | 5/6 |
| Q6P996-4 | Isoform 4 of Pyridoxal-dependent decarboxylase domain-containing protein 1 | 1.5±0.3 | 0.02 | 101.0 | 28.8 | 83.5 | 7 | 3/3 | 2/3 | 1/1 | 1/6 |
| Q8TCT9-5 | Isoform 5 of Minor histocompatibility antigen H13 | 1.5±0.4 | 0.03 | 25.6 | 14.3 | 36.8 | 9 | 1/3 | 3/3 | 1/1 | 4/6 |
| Q16625-5 | Isoform 5 of Occludin | 1.5±0.3 | 0.02 | 24.1 | 35.0 | 23.3 | 9 | 3/3 | 2/3 | 0/1 | 4/6 |
| Q86X29-6 | Isoform 6 of Lipolysis-stimulated lipoprotein receptor | 1.5±0.4 | 0.05 | 26.3 | 22.1 | 54.5 | 8 | 2/3 | 2/3 | 1/1 | 3/6 |
| Q10567-3 | Isoform C of AP-1 complex subunit beta-1 | 1.4±0.3 | 0.04 | 255.6 | 41.2 | 103.5 | 9 | 2/3 | 2/3 | 1/1 | 4/6 |
| O15460-2 | Isoform IIa of Prolyl 4-hydroxylase subunit alpha-2 | 1.7±0.3 | 0.00 | 48.6 | 31.9 | 60.6 | 12 | 3/3 | 3/3 | 1/1 | 5/6 |
| P08727 | Keratin, type I cytoskeletal 19 | 2.1±0.7 | 0.01 | 808.8 | 78.8 | 44.1 | 13 | 3/3 | 3/3 | 1/1 | 6/6 |
| Q96AG4 | Leucine-rich repeat-containing protein 59 | 1.6±0.3 | 0.01 | 169.3 | 65.8 | 34.9 | 11 | 3/3 | 3/3 | 1/1 | 4/6 |
| Q8IVH4 | Methylmalonic aciduria type A protein, mitochondrial | 1.5±0.3 | 0.03 | 36.9 | 23.9 | 46.5 | 8 | 3/3 | 2/3 | 0/1 | 3/6 |
| Q9Y6C9 | Mitochondrial carrier homolog 2 | 1.5±0.3 | 0.02 | 89.1 | 37.6 | 33.3 | 7 | 1/3 | 1/3 | 1/1 | 4/6 |
| Q8IXI1 | Mitochondrial Rho GTPase 2 | 1.4±0.2 | 0.03 | 34.7 | 16.7 | 68.1 | 8 | 0/3 | 2/3 | 1/1 | 5/6 |
| P23368 | NAD-dependent malic enzyme, mitochondrial | 1.4±0.2 | 0.01 | 174.3 | 53.4 | 65.4 | 8 | 2/3 | 2/3 | 1/1 | 3/6 |
| P55160 | Nck-associated protein 1-like | 1.6±0.4 | 0.02 | 12.0 | 3.3 | 128.1 | 9 | 3/3 | 3/3 | 0/1 | 3/6 |
| P45877 | Peptidyl-prolyl cis-trans isomerase C | 2±0.5 | 0.00 | 27.9 | 20.3 | 22.7 | 13 | 3/3 | 3/3 | 1/1 | 6/6 |
| O95571 | Persulfide dioxygenase ETHE1, mitochondrial | 1.6±0.4 | 0.03 | 77.7 | 61.8 | 27.9 | 9 | 2/3 | 3/3 | 0/1 | 4/6 |
| Q9Y446 | Plakophilin-3 | 1.8±0.6 | 0.05 | 142.1 | 33.6 | 87.0 | 10 | 3/3 | 2/3 | 1/1 | 4/6 |
| Q8NBJ5 | Procollagen galactosyltransferase 1 | 1.4±0.2 | 0.00 | 107.7 | 34.9 | 71.6 | 8 | 2/3 | 1/3 | 1/1 | 4/6 |
| E7ETU9 | Procollagen-lysine,2-oxoglutarate 5-dioxygenase 2 | 1.4±0.3 | 0.03 | 73.5 | 29.3 | 81.1 | 8 | 3/3 | 2/3 | 0/1 | 3/6 |
| P61289 | Proteasome activator complex subunit 3 | 1.4±0.3 | 0.01 | 70.8 | 47.2 | 29.5 | 8 | 2/3 | 2/3 | 1/1 | 3/6 |
| P07237 | Protein disulfide-isomerase | 1.4±0.3 | 0.03 | 759.1 | 76.8 | 57.1 | 8 | 2/3 | 3/3 | 1/1 | 2/6 |
| Q96C01 | Protein FAM136A | 1.5±0.4 | 0.04 | 95.5 | 79.7 | 15.6 | 8 | 2/3 | 1/3 | 1/1 | 4/6 |
| Q92520 | Protein FAM3C | 1.5±0.2 | 0.00 | 102.2 | 64.8 | 24.7 | 12 | 3/3 | 3/3 | 1/1 | 5/6 |
| P31949 | Protein S100-A11 | 1.7±0.5 | 0.02 | 226.2 | 56.2 | 11.7 | 11 | 2/3 | 3/3 | 1/1 | 5/6 |
| O94855 | Protein transport protein Sec24D | 1.3±0.2 | 0.02 | 88.0 | 24.1 | 112.9 | 7 | 2/3 | 2/3 | 0/1 | 3/6 |
| P00491 | Purine nucleoside phosphorylase | 1.5±0.4 | 0.02 | 109.7 | 67.8 | 32.1 | 8 | 2/3 | 3/3 | 0/1 | 3/6 |
| P98171 | Rho GTPase-activating protein 4 | 1.4±0.2 | 0.02 | 81.4 | 22.0 | 105.0 | 7 | 1/3 | 2/3 | 0/1 | 4/6 |
| Q9Y5M8 | Signal recognition particle receptor subunit beta | 1.4±0.2 | 0.01 | 109.0 | 48.7 | 29.7 | 7 | 2/3 | 1/3 | 0/1 | 4/6 |
| Q99523 | Sortilin | 1.4±0.3 | 0.04 | 31.6 | 9.3 | 92.0 | 8 | 1/3 | 2/3 | 1/1 | 4/6 |
| O95470 | Sphingosine-1-phosphate lyase 1 | 1.5±0.3 | 0.02 | 139.2 | 52.5 | 63.5 | 9 | 1/3 | 2/3 | 1/1 | 5/6 |
| Q92791 | Synaptonemal complex protein SC65 | 1.5±0.3 | 0.03 | 9.0 | 7.3 | 50.3 | 8 | 1/3 | 2/3 | 1/1 | 4/6 |
| Q15833 | Syntaxin-binding protein 2 | 1.6±0.3 | 0.01 | 118.1 | 45.4 | 66.4 | 11 | 1/3 | 3/3 | 1/1 | 6/6 |
| Q9UGI8 | Testin | 1.4±0.1 | 0.00 | 178.0 | 63.0 | 48.0 | 9 | 2/3 | 2/3 | 1/1 | 4/6 |
| P51580 | Thiopurine S-methyltransferase | 1.6±0.4 | 0.02 | 22.1 | 23.3 | 28.2 | 9 | 3/3 | 1/3 | 1/1 | 4/6 |
| O14530 | Thioredoxin domain-containing protein 9 | 1.4±0.2 | 0.01 | 21.4 | 20.8 | 26.5 | 9 | 3/3 | 2/3 | 0/1 | 4/6 |
| P26639 | Threonine--tRNA ligase, cytoplasmic | 1.3±0.2 | 0.03 | 222.6 | 45.0 | 83.4 | 7 | 1/3 | 1/3 | 1/1 | 4/6 |
| C9JA28 | Translocon-associated protein subunit gamma | 1.5±0.3 | 0.04 | 23.0 | 19.0 | 20.1 | 7 | 1/3 | 1/3 | 1/1 | 4/6 |
| Q99805 | Transmembrane 9 superfamily member 2 | 1.3±0.2 | 0.02 | 47.3 | 14.3 | 75.7 | 7 | 2/3 | 1/3 | 1/1 | 3/6 |
| Q9HD45 | Transmembrane 9 superfamily member 3 | 1.4±0.3 | 0.05 | 32.6 | 11.0 | 67.8 | 8 | 2/3 | 2/3 | 1/1 | 3/6 |
| Q9UM00 | Transmembrane and coiled-coil domain-containing protein 1 | 1.5±0.4 | 0.04 | 26.1 | 28.2 | 21.2 | 8 | 1/3 | 2/3 | 1/1 | 4/6 |
| Q9Y3Q3 | Transmembrane emp24 domain-containing protein 3 | 1.7±0.4 | 0.01 | 17.9 | 18.4 | 24.8 | 10 | 2/3 | 2/3 | 1/1 | 5/6 |
| Q9HC07 | Transmembrane protein 165 | 1.9±0.5 | 0.01 | 7.3 | 10.2 | 34.9 | 12 | 2/3 | 3/3 | 1/1 | 6/6 |
| P43405 | Tyrosine-protein kinase SYK | 1.6±0.3 | 0.00 | 110.7 | 41.9 | 72.0 | 11 | 2/3 | 3/3 | 0/1 | 6/6 |
| P29350 | Tyrosine-protein phosphatase non-receptor type 6 | 1.6±0.2 | 0.00 | 115.4 | 31.6 | 67.5 | 12 | 2/3 | 3/3 | 1/1 | 6/6 |
| A0A096LNZ9 | Ubiquitin-like protein ISG15 (Fragment) | 1.9±0.6 | 0.02 | 49.1 | 42.7 | 15.6 | 11 | 2/3 | 3/3 | 1/1 | 5/6 |
| P21796 | Voltage-dependent anion-selective channel protein 1 | 1.5±0.3 | 0.04 | 978.2 | 79.2 | 30.8 | 7 | 3/3 | 1/3 | 1/1 | 2/6 |
| P21283 | V-type proton ATPase subunit C 1 | 1.4±0.3 | 0.04 | 40.9 | 28.0 | 43.9 | 7 | 0/3 | 2/3 | 1/1 | 4/6 |
| A0A087WTX2 | Zinc transporter SLC39A7 | 2±0.7 | 0.04 | 18.7 | 6.3 | 46.4 | 11 | 2/3 | 2/3 | 1/1 | 6/6 |
