# Supplementary material for: Identification and validation of differentially expressed proteins in epithelial ovarian cancers using quantitative proteomics
Source: Oncotarget. 2016 Nov 4;7(50):83187–99. doi: 10.18632/oncotarget.13077 (PMC5347761; doi:10.18632/oncotarget.13077)
Supplement: Supplementary file 3 [file oncotarget-07-83187-s003.docx]

Table S3. Down regulated proteins in ovarian cancer tissues compared with normal ovarian tissues.

| Accession | Description | Mean±SD | *p*-value | Score | Coverage | MW [kDa] | Number of patients (n=13) | proportion in mucinous adenocarcinoma(n=3) | proportion in clear cell carcinoma(n=3) | proportion in endometrial adenocarcinoma(n=1) | proportion in serous adenocarcinoma  (n=6) |
| --- | --- | --- | --- | --- | --- | --- | --- | --- | --- | --- | --- |
| Q9NRX4 | 14 kDa phosphohistidine phosphatase | 0.5±0.1 | 0.00 | 41.0 | 43.2 | 13.8 | 13 | 3/3 | 3/3 | 1/1 | 6/6 |
| P46952 | 3-hydroxyanthranilate 3,4-dioxygenase | 0.7±0.1 | 0.00 | 8.5 | 15.4 | 32.5 | 9 | 1/3 | 3/3 | 1/1 | 4/6 |
| Q9BUT1 | 3-hydroxybutyrate dehydrogenase type 2 | 0.6±0.2 | 0.00 | 61.6 | 46.5 | 26.7 | 9 | 1/3 | 2/3 | 1/1 | 5/6 |
| Q06136 | 3-ketodihydrosphingosine reductase | 0.6±0.1 | 0.00 | 33.8 | 19.9 | 36.2 | 12 | 2/3 | 3/3 | 1/1 | 6/6 |
| P25325 | 3-mercaptopyruvate sulfurtransferase | 0.6±0.2 | 0.00 | 83.2 | 48.5 | 33.2 | 12 | 2/3 | 3/3 | 1/1 | 6/6 |
| P49189 | 4-trimethylaminobutyraldehyde dehydrogenase | 0.7±0.1 | 0.00 | 257.0 | 52.8 | 53.8 | 8 | 2/3 | 2/3 | 0/1 | 4/6 |
| Q8TCD5 | 5'(3')-deoxyribonucleotidase, cytosolic type | 0.7±0.1 | 0.00 | 51.7 | 51.7 | 23.4 | 7 | 2/3 | 1/3 | 1/1 | 3/6 |
| P62891 | 60S ribosomal protein L39 | 0.4±0.2 | 0.00 | 15.7 | 19.6 | 6.4 | 9 | 3/3 | 1/3 | 0/1 | 5/6 |
| O95336 | 6-phosphogluconolactonase | 0.7±0.1 | 0.00 | 118.6 | 66.3 | 27.5 | 9 | 2/3 | 1/3 | 0/1 | 6/6 |
| Q9H6R3 | Acyl-CoA synthetase short-chain family member 3, mitochondrial | 0.7±0.2 | 0.00 | 78.3 | 34.6 | 74.7 | 9 | 2/3 | 2/3 | 1/1 | 4/6 |
| P00568 | Adenylate kinase isoenzyme 1 | 0.4±0.1 | 0.00 | 124.0 | 77.8 | 21.6 | 13 | 3/3 | 3/3 | 1/1 | 6/6 |
| Q15847 | Adipogenesis regulatory factor | 0.4±0.3 | 0.01 | 91.2 | 80.3 | 7.8 | 12 | 3/3 | 3/3 | 1/1 | 5/6 |
| Q10588 | ADP-ribosyl cyclase/cyclic ADP-ribose hydrolase 2 | 0.5±0.2 | 0.00 | 29.6 | 15.7 | 35.7 | 11 | 2/3 | 3/3 | 1/1 | 5/6 |
| O43488 | Aflatoxin B1 aldehyde reductase member 2 | 0.7±0.1 | 0.00 | 110.1 | 41.0 | 39.6 | 11 | 2/3 | 3/3 | 1/1 | 6/6 |
| P11766 | Alcohol dehydrogenase class-3 | 0.5±0.1 | 0.00 | 274.8 | 43.3 | 39.7 | 13 | 3/3 | 3/3 | 1/1 | 6/6 |
| Q06278 | Aldehyde oxidase | 0.5±0.2 | 0.00 | 34.5 | 12.6 | 147.8 | 12 | 3/3 | 3/3 | 1/1 | 5/6 |
| Q96C23 | Aldose 1-epimerase | 0.7±0.1 | 0.00 | 80.7 | 45.0 | 37.7 | 7 | 1/3 | 2/3 | 0/1 | 4/6 |
| P15121 | Aldose reductase | 0.5±0.1 | 0.00 | 171.1 | 73.4 | 35.8 | 13 | 3/3 | 3/3 | 1/1 | 6/6 |
| Q96IU4 | Alpha/beta hydrolase domain-containing protein 14B | 0.7±0.1 | 0.00 | 85.4 | 53.3 | 22.3 | 9 | 2/3 | 1/3 | 1/1 | 5/6 |
| P35611 | Alpha-adducin | 0.7±0.1 | 0.00 | 122.9 | 31.2 | 80.9 | 7 | 1/3 | 3/3 | 0/1 | 3/6 |
| Q9UDR5 | Alpha-aminoadipic semialdehyde synthase, mitochondrial | 0.6±0.1 | 0.00 | 83.6 | 28.3 | 102.1 | 12 | 3/3 | 3/3 | 1/1 | 5/6 |
| P27338 | Amine oxidase [flavin-containing] B | 0.5±0.1 | 0.00 | 279.0 | 55.4 | 58.7 | 13 | 3/3 | 3/3 | 1/1 | 6/6 |
| Q8IV38 | Ankyrin repeat and MYND domain-containing protein 2 | 0.7±0.1 | 0.00 | 31.4 | 19.1 | 49.3 | 9 | 3/3 | 2/3 | 1/1 | 3/6 |
| I6L894 | Ankyrin-2 | 0.6±0.2 | 0.00 | 97.3 | 5.7 | 430.0 | 10 | 1/3 | 2/3 | 1/1 | 6/6 |
| P07355 | Annexin A2 | 0.6±0.2 | 0.00 | 1416.1 | 88.2 | 38.6 | 11 | 2/3 | 3/3 | 1/1 | 5/6 |
| P08758 | Annexin A5 | 0.6±0.2 | 0.00 | 487.1 | 76.6 | 35.9 | 12 | 2/3 | 3/3 | 1/1 | 6/6 |
| Q7Z6K5 | Arpin | 0.7±0.1 | 0.00 | 33.4 | 41.2 | 24.9 | 7 | 2/3 | 1/3 | 1/1 | 3/6 |
| Q9ULA0 | Aspartyl aminopeptidase | 0.6±0.1 | 0.00 | 172.2 | 46.1 | 52.4 | 13 | 3/3 | 3/3 | 1/1 | 6/6 |
| A0A0A0MSU4 | ATP-binding cassette sub-family A member 8 | 0.6±0.1 | 0.00 | 17.9 | 3.8 | 182.9 | 13 | 3/3 | 3/3 | 1/1 | 6/6 |
| Q8IW45 | ATP-dependent (S)-NAD(P)H-hydrate dehydratase | 0.7±0.1 | 0.00 | 107.8 | 55.3 | 36.6 | 10 | 3/3 | 1/3 | 1/1 | 5/6 |
| P08237 | ATP-dependent 6-phosphofructokinase, muscle type | 0.7±0.2 | 0.00 | 209.5 | 43.9 | 85.1 | 9 | 2/3 | 2/3 | 1/1 | 4/6 |
| H0Y5B0 | Band 4.1-like protein 2 (Fragment) | 0.5±0.1 | 0.00 | 83.1 | 47.9 | 42.1 | 13 | 3/3 | 3/3 | 1/1 | 6/6 |
| O75531 | Barrier-to-autointegration factor | 0.4±0.1 | 0.00 | 167.9 | 80.9 | 10.1 | 13 | 3/3 | 3/3 | 1/1 | 6/6 |
| Q13425 | Beta-2-syntrophin | 0.7±0.1 | 0.00 | 75.0 | 29.1 | 57.9 | 10 | 2/3 | 3/3 | 1/1 | 4/6 |
| Q16585 | Beta-sarcoglycan | 0.6±0.2 | 0.01 | 11.6 | 9.4 | 34.8 | 11 | 2/3 | 2/3 | 1/1 | 6/6 |
| Q8NFC6 | Biorientation of chromosomes in cell division protein 1-like 1 | 0.7±0.1 | 0.00 | 23.2 | 2.8 | 330.3 | 8 | 3/3 | 3/3 | 0/1 | 2/6 |
| P55287 | Cadherin-11 | 0.5±0.1 | 0.00 | 42.4 | 14.8 | 87.9 | 12 | 3/3 | 2/3 | 1/1 | 6/6 |
| Q9H9S4 | Calcium-binding protein 39-like | 0.6±0.3 | 0.03 | 9.8 | 8.0 | 39.1 | 8 | 2/3 | 1/3 | 0/1 | 5/6 |
| P17612 | cAMP-dependent protein kinase catalytic subunit alpha | 0.4±0.1 | 0.00 | 136.6 | 46.4 | 40.6 | 13 | 3/3 | 3/3 | 1/1 | 6/6 |
| P22694 | cAMP-dependent protein kinase catalytic subunit beta | 0.6±0.1 | 0.00 | 83.4 | 41.6 | 40.6 | 12 | 3/3 | 3/3 | 1/1 | 5/6 |
| P31321 | cAMP-dependent protein kinase type I-beta regulatory subunit | 0.7±0.2 | 0.01 | 29.9 | 18.1 | 43.0 | 7 | 2/3 | 0/3 | 1/1 | 4/6 |
| P13861 | cAMP-dependent protein kinase type II-alpha regulatory subunit | 0.7±0.1 | 0.00 | 205.4 | 61.1 | 45.5 | 12 | 2/3 | 3/3 | 1/1 | 6/6 |
| P31323 | cAMP-dependent protein kinase type II-beta regulatory subunit | 0.3±0.1 | 0.00 | 170.4 | 64.1 | 46.3 | 13 | 3/3 | 3/3 | 1/1 | 6/6 |
| O75828 | Carbonyl reductase [NADPH] 3 | 0.6±0.2 | 0.00 | 54.3 | 39.7 | 30.8 | 11 | 3/3 | 2/3 | 1/1 | 5/6 |
| P14384 | Carboxypeptidase M | 0.7±0.2 | 0.02 | 17.6 | 8.4 | 50.5 | 9 | 3/3 | 1/3 | 1/1 | 4/6 |
| Q03135 | Caveolin-1 | 0.5±0.2 | 0.00 | 77.3 | 45.5 | 20.5 | 12 | 3/3 | 3/3 | 1/1 | 5/6 |
| Q96F85 | CB1 cannabinoid receptor-interacting protein 1 | 0.4±0.1 | 0.00 | 20.8 | 31.1 | 18.6 | 13 | 3/3 | 3/3 | 1/1 | 6/6 |
| E9PNW4 | CD59 glycoprotein | 0.5±0.1 | 0.00 | 67.8 | 33.3 | 12.0 | 13 | 3/3 | 3/3 | 1/1 | 6/6 |
| Q9H3Q1 | Cdc42 effector protein 4 | 0.6±0.2 | 0.00 | 41.7 | 43.5 | 38.0 | 11 | 3/3 | 2/3 | 0/1 | 6/6 |
| P41208 | Centrin-2 | 0.7±0.1 | 0.00 | 50.9 | 69.2 | 19.7 | 10 | 3/3 | 3/3 | 0/1 | 4/6 |
| Q7Z7K6 | Centromere protein V | 0.5±0.2 | 0.00 | 48.7 | 49.5 | 29.9 | 12 | 3/3 | 3/3 | 1/1 | 5/6 |
| O15247 | Chloride intracellular channel protein 2 | 0.6±0.1 | 0.00 | 15.9 | 26.3 | 28.3 | 12 | 3/3 | 2/3 | 1/1 | 6/6 |
| J3KS05 | Chromobox protein homolog 1 (Fragment) | 0.5±0.1 | 0.00 | 180.7 | 59.5 | 20.0 | 13 | 3/3 | 3/3 | 1/1 | 6/6 |
| Q9Y281 | Cofilin-2 | 0.5±0.4 | 0.04 | 180.5 | 78.9 | 18.7 | 12 | 3/3 | 3/3 | 1/1 | 5/6 |
| Q9H0W5 | Coiled-coil domain-containing protein 8 | 0.6±0.2 | 0.01 | 5.9 | 6.3 | 59.3 | 9 | 3/3 | 2/3 | 0/1 | 4/6 |
| Q14011 | Cold-inducible RNA-binding protein | 0.6±0.2 | 0.00 | 65.9 | 39.5 | 18.6 | 12 | 3/3 | 3/3 | 1/1 | 5/6 |
| Q9BXR6 | Complement factor H-related protein 5 | 0.6±0.2 | 0.01 | 63.9 | 28.3 | 64.4 | 9 | 2/3 | 2/3 | 1/1 | 4/6 |
| P42773 | Cyclin-dependent kinase 4 inhibitor C | 0.6±0.1 | 0.00 | 5.9 | 14.3 | 18.1 | 11 | 1/3 | 3/3 | 1/1 | 6/6 |
| P21399 | Cytoplasmic aconitate hydratase | 0.7±0.2 | 0.01 | 257.2 | 50.4 | 98.3 | 8 | 0/3 | 1/3 | 1/1 | 6/6 |
| O75891 | Cytosolic 10-formyltetrahydrofolate dehydrogenase | 0.5±0.2 | 0.01 | 30.2 | 8.8 | 98.8 | 7 | 1/3 | 1/3 | 1/1 | 4/6 |
| Q8NFI3 | Cytosolic endo-beta-N-acetylglucosaminidase | 0.7±0.1 | 0.00 | 5.7 | 1.6 | 83.9 | 8 | 3/3 | 2/3 | 1/1 | 3/6 |
| Q9UKG1 | DCC-interacting protein 13-alpha | 0.7±0.1 | 0.00 | 84.2 | 33.4 | 79.6 | 8 | 1/3 | 1/3 | 1/1 | 5/6 |
| Q13268 | Dehydrogenase/reductase SDR family member 2, mitochondrial | 0.4±0.2 | 0.00 | 58.7 | 33.9 | 29.9 | 12 | 3/3 | 3/3 | 1/1 | 5/6 |
| P13716 | Delta-aminolevulinic acid dehydratase | 0.6±0.2 | 0.00 | 62.3 | 37.6 | 36.3 | 10 | 2/3 | 2/3 | 1/1 | 5/6 |
| Q92629 | Delta-sarcoglycan | 0.5±0.2 | 0.00 | 39.6 | 36.0 | 32.1 | 11 | 2/3 | 2/3 | 1/1 | 6/6 |
| Q9BW61 | DET1- and DDB1-associated protein 1 | 0.7±0.2 | 0.02 | 9.9 | 18.6 | 11.8 | 8 | 3/3 | 1/3 | 0/1 | 4/6 |
| A6NML8 | Diaphanous homolog 2 (Drosophila), isoform CRA_c | 0.7±0.2 | 0.00 | 26.9 | 8.4 | 124.8 | 9 | 1/3 | 3/3 | 1/1 | 4/6 |
| P09417 | Dihydropteridine reductase | 0.7±0.1 | 0.00 | 73.3 | 58.2 | 25.8 | 7 | 1/3 | 1/3 | 1/1 | 4/6 |
| Q12882 | Dihydropyrimidine dehydrogenase [NADP(+)] | 0.6±0.1 | 0.00 | 92.2 | 29.4 | 111.3 | 11 | 3/3 | 2/3 | 1/1 | 5/6 |
| P27487 | Dipeptidyl peptidase 4 | 0.6±0.2 | 0.00 | 60.0 | 18.7 | 88.2 | 10 | 2/3 | 1/3 | 1/1 | 6/6 |
| O95989 | Diphosphoinositol polyphosphate phosphohydrolase 1 | 0.7±0.1 | 0.00 | 31.8 | 44.8 | 19.5 | 11 | 3/3 | 2/3 | 1/1 | 5/6 |
| Q9NZJ9 | Diphosphoinositol polyphosphate phosphohydrolase 2 | 0.5±0.2 | 0.00 | 17.4 | 23.3 | 20.3 | 11 | 3/3 | 3/3 | 0/1 | 5/6 |
| Q92466 | DNA damage-binding protein 2 | 0.7±0.2 | 0.00 | 9.4 | 6.3 | 47.8 | 11 | 3/3 | 3/3 | 1/1 | 4/6 |
| O75937 | DnaJ homolog subfamily C member 8 | 0.7±0.2 | 0.01 | 115.9 | 53.0 | 29.8 | 10 | 3/3 | 3/3 | 0/1 | 4/6 |
| F5GXX5 | Dolichyl-diphosphooligosaccharide--protein glycosyltransferase subunit DAD1 | 0.6±0.1 | 0.00 | 12.5 | 25.9 | 9.5 | 10 | 3/3 | 2/3 | 0/1 | 5/6 |
| Q9Y4X5 | E3 ubiquitin-protein ligase ARIH1 | 0.7±0.1 | 0.00 | 28.7 | 21.2 | 64.1 | 7 | 1/3 | 1/3 | 0/1 | 5/6 |
| Q6UWR7 | Ectonucleotide pyrophosphatase/phosphodiesterase family member 6 | 0.4±0.2 | 0.00 | 130.4 | 37.3 | 50.2 | 12 | 3/3 | 3/3 | 1/1 | 5/6 |
| Q9NZN4 | EH domain-containing protein 2 | 0.3±0.2 | 0.00 | 313.5 | 66.7 | 61.1 | 13 | 3/3 | 3/3 | 1/1 | 6/6 |
| Q9NZN3 | EH domain-containing protein 3 | 0.7±0.1 | 0.00 | 98.2 | 39.4 | 60.8 | 8 | 1/3 | 1/3 | 1/1 | 5/6 |
| Q6ICJ4 | Em:AP000351.3 protein | 0.5±0.3 | 0.00 | 15.7 | 22.2 | 25.9 | 10 | 2/3 | 2/3 | 1/1 | 5/6 |
| P50402 | Emerin | 0.7±0.1 | 0.00 | 80.5 | 55.1 | 29.0 | 10 | 1/3 | 3/3 | 1/1 | 5/6 |
| Q9NT22 | EMILIN-3 | 0.4±0.2 | 0.00 | 10.0 | 1.7 | 82.6 | 12 | 3/3 | 3/3 | 1/1 | 5/6 |
| D6RA00 | Enolase-phosphatase E1 | 0.6±0.2 | 0.00 | 25.0 | 42.8 | 19.2 | 10 | 2/3 | 2/3 | 1/1 | 5/6 |
| Q7L775 | EPM2A-interacting protein 1 | 0.7±0.1 | 0.00 | 36.8 | 14.3 | 70.3 | 10 | 3/3 | 3/3 | 0/1 | 4/6 |
| P07099 | Epoxide hydrolase 1 | 0.7±0.2 | 0.03 | 294.2 | 58.9 | 52.9 | 9 | 1/3 | 3/3 | 1/1 | 4/6 |
| E9PEH6 | Epsilon-sarcoglycan | 0.6±0.3 | 0.01 | 18.6 | 17.7 | 45.0 | 10 | 1/3 | 2/3 | 1/1 | 6/6 |
| O75477 | Erlin-1 | 0.7±0.1 | 0.00 | 151.4 | 50.0 | 38.9 | 9 | 1/3 | 3/3 | 1/1 | 4/6 |
| O94905 | Erlin-2 | 0.5±0.1 | 0.00 | 271.6 | 67.3 | 37.8 | 13 | 3/3 | 3/3 | 1/1 | 6/6 |
| P47813 | Eukaryotic translation initiation factor 1A, X-chromosomal | 0.7±0.1 | 0.00 | 63.4 | 43.1 | 16.5 | 11 | 3/3 | 3/3 | 0/1 | 5/6 |
| Q16658 | Fascin | 0.7±0.2 | 0.01 | 268.9 | 60.9 | 54.5 | 10 | 2/3 | 2/3 | 1/1 | 5/6 |
| P58012 | Forkhead box protein L2 | 0.6±0.3 | 0.04 | 9.1 | 8.2 | 38.7 | 7 | 1/3 | 2/3 | 0/1 | 4/6 |
| Q96PY5 | Formin-like protein 2 | 0.7±0.1 | 0.00 | 49.1 | 11.7 | 123.2 | 11 | 3/3 | 2/3 | 0/1 | 6/6 |
| Q14192 | Four and a half LIM domains protein 2 | 0.7±0.2 | 0.04 | 164.3 | 69.2 | 32.2 | 10 | 2/3 | 1/3 | 1/1 | 6/6 |
| Q9H479 | Fructosamine-3-kinase | 0.7±0.1 | 0.00 | 37.0 | 27.2 | 35.1 | 10 | 2/3 | 2/3 | 1/1 | 5/6 |
| P16930 | Fumarylacetoacetase | 0.8±0.2 | 0.03 | 143.9 | 53.2 | 46.3 | 9 | 1/3 | 3/3 | 1/1 | 5/6 |
| Q9BQS8 | FYVE and coiled-coil domain-containing protein 1 | 0.6±0.1 | 0.00 | 118.8 | 21.0 | 166.9 | 9 | 1/3 | 2/3 | 1/1 | 5/6 |
| P09382 | Galectin-1 | 0.6±0.2 | 0.00 | 908.7 | 82.2 | 14.7 | 11 | 3/3 | 3/3 | 1/1 | 4/6 |
| O95479 | GDH/6PGL endoplasmic bifunctional protein | 0.7±0.2 | 0.01 | 236.2 | 49.2 | 88.8 | 8 | 2/3 | 3/3 | 1/1 | 2/6 |
| Q8TDQ7 | Glucosamine-6-phosphate isomerase 2 | 0.6±0.1 | 0.00 | 47.0 | 49.6 | 31.1 | 11 | 2/3 | 2/3 | 1/1 | 6/6 |
| P11413 | Glucose-6-phosphate 1-dehydrogenase | 0.7±0.1 | 0.00 | 171.8 | 55.3 | 59.2 | 8 | 1/3 | 1/3 | 1/1 | 5/6 |
| P09488 | Glutathione S-transferase Mu 1 | 0.2±0.1 | 0.00 | 57.0 | 40.8 | 25.7 | 13 | 3/3 | 3/3 | 1/1 | 6/6 |
| P28161 | Glutathione S-transferase Mu 2 | 0.2±0 | 0.00 | 100.7 | 61.9 | 25.7 | 13 | 3/3 | 3/3 | 1/1 | 6/6 |
| P21266 | Glutathione S-transferase Mu 3 | 0.4±0.1 | 0.00 | 79.3 | 66.7 | 26.5 | 13 | 3/3 | 3/3 | 1/1 | 6/6 |
| P46439 | Glutathione S-transferase Mu 5 | 0.3±0 | 0.00 | 36.1 | 27.5 | 25.7 | 8 | 2/3 | 3/3 | 1/1 | 2/6 |
| P30711 | Glutathione S-transferase theta-1 | 0.4±0.2 | 0.00 | 19.2 | 21.3 | 27.3 | 12 | 3/3 | 3/3 | 1/1 | 5/6 |
| Q8TBN0 | Guanine nucleotide exchange factor for Rab-3A | 0.5±0.2 | 0.00 | 6.6 | 5.8 | 42.6 | 11 | 3/3 | 2/3 | 1/1 | 5/6 |
| P63096 | Guanine nucleotide-binding protein G(i) subunit alpha-1 | 0.7±0.2 | 0.01 | 182.9 | 37.3 | 40.3 | 8 | 2/3 | 1/3 | 1/1 | 4/6 |
| P59768 | Guanine nucleotide-binding protein G(I)/G(S)/G(O) subunit gamma-2 | 0.7±0.2 | 0.01 | 19.0 | 52.1 | 7.8 | 9 | 2/3 | 1/3 | 1/1 | 5/6 |
| P50150 | Guanine nucleotide-binding protein G(I)/G(S)/G(O) subunit gamma-4 | 0.3±0.2 | 0.00 | 6.0 | 9.3 | 8.4 | 13 | 3/3 | 3/3 | 1/1 | 6/6 |
| P62873 | Guanine nucleotide-binding protein G(I)/G(S)/G(T) subunit beta-1 | 0.6±0.1 | 0.00 | 291.7 | 44.7 | 37.4 | 13 | 3/3 | 3/3 | 1/1 | 6/6 |
| P62879 | Guanine nucleotide-binding protein G(I)/G(S)/G(T) subunit beta-2 | 0.7±0.1 | 0.00 | 291.6 | 46.2 | 37.3 | 11 | 2/3 | 3/3 | 1/1 | 5/6 |
| Q14344 | Guanine nucleotide-binding protein subunit alpha-13 | 0.7±0.2 | 0.01 | 96.3 | 29.2 | 44.0 | 10 | 2/3 | 1/3 | 1/1 | 6/6 |
| Q9H0R4 | Haloacid dehalogenase-like hydrolase domain-containing protein 2 | 0.6±0.1 | 0.00 | 37.0 | 30.9 | 28.5 | 11 | 3/3 | 2/3 | 1/1 | 5/6 |
| O43301 | Heat shock 70 kDa protein 12A | 0.4±0.1 | 0.00 | 99.6 | 36.6 | 74.9 | 13 | 3/3 | 3/3 | 1/1 | 6/6 |
| Q5JX83 | Heat shock 70 kDa protein 12B | 0.7±0.1 | 0.00 | 35.5 | 18.2 | 66.8 | 10 | 1/3 | 2/3 | 1/1 | 6/6 |
| P08107 | Heat shock 70 kDa protein 1A/1B | 0.7±0.1 | 0.00 | 1059.3 | 74.1 | 70.0 | 9 | 3/3 | 3/3 | 0/1 | 4/6 |
| P09601 | Heme oxygenase 1 | 0.5±0.2 | 0.00 | 70.6 | 43.1 | 32.8 | 12 | 3/3 | 2/3 | 1/1 | 6/6 |
| Q9NRV9 | Heme-binding protein 1 | 0.7±0.1 | 0.00 | 69.8 | 74.1 | 21.1 | 11 | 2/3 | 3/3 | 1/1 | 5/6 |
| Q9Y3E1 | Hepatoma-derived growth factor-related protein 3 | 0.5±0.2 | 0.00 | 68.6 | 34.5 | 22.6 | 12 | 3/3 | 3/3 | 1/1 | 5/6 |
| Q13151 | Heterogeneous nuclear ribonucleoprotein A0 | 0.7±0.1 | 0.00 | 178.5 | 48.9 | 30.8 | 11 | 3/3 | 3/3 | 0/1 | 5/6 |
| P51991 | Heterogeneous nuclear ribonucleoprotein A3 | 0.7±0.1 | 0.00 | 622.8 | 52.4 | 39.6 | 7 | 2/3 | 3/3 | 0/1 | 2/6 |
| A0A087WUK2 | Heterogeneous nuclear ribonucleoprotein D-like | 0.7±0.2 | 0.02 | 174.7 | 33.6 | 40.0 | 11 | 3/3 | 3/3 | 0/1 | 5/6 |
| O00479 | High mobility group nucleosome-binding domain-containing protein 4 | 0.6±0.2 | 0.00 | 50.9 | 58.9 | 9.5 | 11 | 2/3 | 3/3 | 1/1 | 5/6 |
| Q5JSK7 | High mobility group nucleosome-binding domain-containing protein 5 (Fragment) | 0.4±0.2 | 0.00 | 20.1 | 53.9 | 9.9 | 13 | 3/3 | 3/3 | 1/1 | 6/6 |
| P09429 | High mobility group protein B1 | 0.6±0.3 | 0.03 | 451.3 | 69.8 | 24.9 | 10 | 3/3 | 3/3 | 0/1 | 4/6 |
| P26583 | High mobility group protein B2 | 0.6±0.2 | 0.00 | 217.3 | 68.9 | 24.0 | 10 | 2/3 | 3/3 | 1/1 | 4/6 |
| Q92522 | Histone H1x | 0.6±0.3 | 0.01 | 86.0 | 41.8 | 22.5 | 11 | 2/3 | 3/3 | 1/1 | 5/6 |
| P0C0S5 | Histone H2A.Z | 0.7±0.2 | 0.01 | 158.2 | 35.9 | 13.5 | 8 | 2/3 | 3/3 | 0/1 | 3/6 |
| K7EK07 | Histone H3 (Fragment) | 0.5±0.3 | 0.00 | 172.5 | 72.0 | 14.9 | 11 | 2/3 | 3/3 | 1/1 | 5/6 |
| P50502 | Hsc70-interacting protein | 0.5±0.1 | 0.00 | 181.5 | 42.3 | 41.3 | 13 | 3/3 | 3/3 | 1/1 | 6/6 |
| Q6YN16 | Hydroxysteroid dehydrogenase-like protein 2 | 0.7±0.1 | 0.00 | 134.0 | 51.7 | 45.4 | 9 | 1/3 | 3/3 | 0/1 | 5/6 |
| Q13308 | Inactive tyrosine-protein kinase 7 | 0.7±0.1 | 0.00 | 187.4 | 34.3 | 118.3 | 8 | 2/3 | 2/3 | 0/1 | 4/6 |
| P24593 | Insulin-like growth factor-binding protein 5 | 0.7±0.2 | 0.04 | 13.4 | 15.4 | 30.6 | 8 | 2/3 | 2/3 | 0/1 | 4/6 |
| P08648 | Integrin alpha-5 | 0.7±0.2 | 0.04 | 30.3 | 9.4 | 114.5 | 8 | 1/3 | 1/3 | 1/1 | 5/6 |
| Q8IU81 | Interferon regulatory factor 2-binding protein 1 | 0.7±0.1 | 0.00 | 37.3 | 17.0 | 61.6 | 11 | 3/3 | 2/3 | 1/1 | 5/6 |
| H7C5G1 | Isoamyl acetate-hydrolyzing esterase 1 homolog (Fragment) | 0.7±0.1 | 0.00 | 66.7 | 45.6 | 25.2 | 8 | 1/3 | 2/3 | 0/1 | 6/6 |
| O75874 | Isocitrate dehydrogenase [NADP] cytoplasmic | 0.6±0.1 | 0.00 | 386.5 | 72.2 | 46.6 | 10 | 2/3 | 2/3 | 0/1 | 6/6 |
| Q9UEY8-2 | Isoform 1 of Gamma-adducin | 0.6±0.1 | 0.00 | 102.3 | 30.0 | 75.6 | 12 | 3/3 | 3/3 | 1/1 | 5/6 |
| P51178-2 | Isoform 2 of 1-phosphatidylinositol 4,5-bisphosphate phosphodiesterase delta-1 | 0.6±0.1 | 0.00 | 36.5 | 18.8 | 88.1 | 12 | 3/3 | 2/3 | 1/1 | 6/6 |
| P21589-2 | Isoform 2 of 5'-nucleotidase | 0.4±0.1 | 0.00 | 192.8 | 50.4 | 57.9 | 13 | 3/3 | 3/3 | 1/1 | 6/6 |
| P49419-2 | Isoform 2 of Alpha-aminoadipic semialdehyde dehydrogenase | 0.7±0.2 | 0.01 | 393.7 | 67.1 | 55.3 | 8 | 2/3 | 3/3 | 0/1 | 3/6 |
| P58335-2 | Isoform 2 of Anthrax toxin receptor 2 | 0.6±0.1 | 0.00 | 14.7 | 8.0 | 42.9 | 10 | 3/3 | 2/3 | 1/1 | 4/6 |
| Q9Y2J2-2 | Isoform 2 of Band 4.1-like protein 3 | 0.6±0.2 | 0.00 | 75.7 | 21.0 | 96.5 | 8 | 2/3 | 1/3 | 1/1 | 4/6 |
| Q10589-2 | Isoform 2 of Bone marrow stromal antigen 2 | 0.6±0.2 | 0.00 | 34.2 | 14.9 | 18.4 | 10 | 2/3 | 3/3 | 1/1 | 4/6 |
| Q5PSV4-2 | Isoform 2 of Breast cancer metastasis-suppressor 1-like protein | 0.7±0.2 | 0.00 | 9.4 | 12.0 | 32.3 | 10 | 2/3 | 3/3 | 1/1 | 4/6 |
| Q9UDT6-2 | Isoform 2 of CAP-Gly domain-containing linker protein 2 | 0.7±0.1 | 0.00 | 48.3 | 13.7 | 111.7 | 9 | 1/3 | 2/3 | 1/1 | 5/6 |
| Q13740-2 | Isoform 2 of CD166 antigen | 0.5±0.3 | 0.01 | 148.7 | 40.2 | 63.6 | 11 | 3/3 | 3/3 | 1/1 | 4/6 |
| Q7Z6B0-2 | Isoform 2 of Coiled-coil domain-containing protein 91 | 0.7±0.1 | 0.00 | 18.7 | 20.9 | 47.0 | 8 | 1/3 | 2/3 | 0/1 | 5/6 |
| Q12860-2 | Isoform 2 of Contactin-1 | 0.7±0.2 | 0.01 | 44.4 | 14.7 | 111.8 | 10 | 1/3 | 2/3 | 1/1 | 6/6 |
| Q9UQ03-2 | Isoform 2 of Coronin-2B | 0.6±0.2 | 0.00 | 32.3 | 23.2 | 54.4 | 11 | 1/3 | 3/3 | 1/1 | 6/6 |
| Q9H0W9-2 | Isoform 2 of Ester hydrolase C11orf54 | 0.7±0.2 | 0.01 | 58.1 | 34.1 | 33.1 | 10 | 2/3 | 3/3 | 1/1 | 4/6 |
| Q9H334-2 | Isoform 2 of Forkhead box protein P1 | 0.6±0.1 | 0.00 | 7.3 | 9.2 | 54.5 | 7 | 1/3 | 1/3 | 0/1 | 5/6 |
| P50440-2 | Isoform 2 of Glycine amidinotransferase, mitochondrial | 0.5±0.1 | 0.00 | 179.4 | 72.1 | 44.9 | 12 | 3/3 | 2/3 | 1/1 | 6/6 |
| Q9HC38-2 | Isoform 2 of Glyoxalase domain-containing protein 4 | 0.7±0.1 | 0.00 | 177.8 | 67.1 | 33.2 | 9 | 2/3 | 2/3 | 0/1 | 5/6 |
| Q7Z4V5-2 | Isoform 2 of Hepatoma-derived growth factor-related protein 2 | 0.6±0.2 | 0.00 | 52.7 | 16.1 | 74.2 | 11 | 2/3 | 3/3 | 1/1 | 5/6 |
| Q9UK53-2 | Isoform 2 of Inhibitor of growth protein 1 | 0.5±0.2 | 0.02 | 5.1 | 7.9 | 31.8 | 8 | 1/3 | 1/3 | 1/1 | 5/6 |
| Q04760-2 | Isoform 2 of Lactoylglutathione lyase | 0.5±0.1 | 0.00 | 121.2 | 65.7 | 19.0 | 13 | 3/3 | 3/3 | 1/1 | 6/6 |
| Q8N6Y2-2 | Isoform 2 of Leucine-rich repeat-containing protein 17 | 0.4±0.2 | 0.00 | 10.0 | 11.8 | 36.3 | 12 | 2/3 | 3/3 | 1/1 | 6/6 |
| P04156-2 | Isoform 2 of Major prion protein | 0.4±0.2 | 0.00 | 7.6 | 4.9 | 26.9 | 12 | 2/3 | 3/3 | 1/1 | 6/6 |
| P84157-2 | Isoform 2 of Matrix-remodeling-associated protein 7 | 0.5±0.3 | 0.02 | 9.2 | 8.8 | 17.5 | 11 | 2/3 | 3/3 | 1/1 | 5/6 |
| Q9UPY8-2 | Isoform 2 of Microtubule-associated protein RP/EB family member 3 | 0.5±0.2 | 0.00 | 16.5 | 12.8 | 30.4 | 11 | 3/3 | 3/3 | 0/1 | 5/6 |
| Q9NYL2-2 | Isoform 2 of Mitogen-activated protein kinase kinase kinase MLT | 0.6±0.2 | 0.00 | 22.3 | 13.9 | 51.5 | 12 | 3/3 | 3/3 | 1/1 | 5/6 |
| Q92859-2 | Isoform 2 of Neogenin | 0.7±0.1 | 0.00 | 21.6 | 5.0 | 154.2 | 10 | 1/3 | 3/3 | 1/1 | 5/6 |
| Q96T66-2 | Isoform 2 of Nicotinamide mononucleotide adenylyltransferase 3 | 0.7±0.2 | 0.02 | 46.7 | 51.2 | 24.1 | 9 | 2/3 | 3/3 | 1/1 | 3/6 |
| Q9BQI9-2 | Isoform 2 of Nuclear receptor-interacting protein 2 | 0.6±0.2 | 0.00 | 6.0 | 10.4 | 29.9 | 12 | 2/3 | 3/3 | 1/1 | 6/6 |
| O75147-2 | Isoform 2 of Obscurin-like protein 1 | 0.7±0.1 | 0.00 | 8.2 | 2.5 | 113.2 | 11 | 3/3 | 3/3 | 0/1 | 5/6 |
| Q9GZU2-2 | Isoform 2 of Paternally-expressed gene 3 protein | 0.7±0.2 | 0.00 | 7.3 | 1.4 | 165.9 | 9 | 2/3 | 2/3 | 1/1 | 4/6 |
| Q9Y680-2 | Isoform 2 of Peptidyl-prolyl cis-trans isomerase FKBP7 | 0.5±0.2 | 0.00 | 61.5 | 53.2 | 25.8 | 11 | 2/3 | 2/3 | 1/1 | 6/6 |
| Q53EL6-2 | Isoform 2 of Programmed cell death protein 4 | 0.7±0.1 | 0.00 | 170.8 | 45.0 | 50.5 | 7 | 2/3 | 2/3 | 0/1 | 3/6 |
| Q9BZG1-2 | Isoform 2 of Ras-related protein Rab-34 | 0.7±0.1 | 0.00 | 29.9 | 24.7 | 28.2 | 9 | 3/3 | 3/3 | 0/1 | 3/6 |
| P18433-2 | Isoform 2 of Receptor-type tyrosine-protein phosphatase alpha | 0.6±0.1 | 0.00 | 25.1 | 9.2 | 89.6 | 11 | 3/3 | 3/3 | 1/1 | 4/6 |
| Q13332-6 | Isoform 2 of Receptor-type tyrosine-protein phosphatase S | 0.6±0.1 | 0.00 | 30.9 | 5.5 | 212.4 | 13 | 3/3 | 3/3 | 1/1 | 6/6 |
| O94788-2 | Isoform 2 of Retinal dehydrogenase 2 | 0.4±0.1 | 0.00 | 32.0 | 19.2 | 53.0 | 13 | 3/3 | 3/3 | 1/1 | 6/6 |
| P05026-2 | Isoform 2 of Sodium/potassium-transporting ATPase subunit beta-1 | 0.7±0.2 | 0.00 | 96.9 | 29.6 | 34.9 | 8 | 2/3 | 2/3 | 0/1 | 4/6 |
| Q01082-3 | Isoform 2 of Spectrin beta chain, non-erythrocytic 1 | 0.7±0.2 | 0.01 | 1364.2 | 73.6 | 251.2 | 8 | 2/3 | 3/3 | 1/1 | 2/6 |
| O60343-2 | Isoform 2 of TBC1 domain family member 4 | 0.6±0.1 | 0.00 | 63.3 | 12.3 | 139.5 | 11 | 3/3 | 2/3 | 1/1 | 5/6 |
| Q9HC78-2 | Isoform 2 of Zinc finger and BTB domain-containing protein 20 | 0.6±0.3 | 0.03 | 12.2 | 7.6 | 73.4 | 10 | 3/3 | 3/3 | 1/1 | 3/6 |
| Q02952-3 | Isoform 3 of A-kinase anchor protein 12 | 0.3±0.1 | 0.00 | 247.9 | 30.7 | 180.9 | 13 | 3/3 | 3/3 | 1/1 | 6/6 |
| Q4KMQ2-3 | Isoform 3 of Anoctamin-6 | 0.5±0.1 | 0.00 | 40.0 | 16.0 | 103.9 | 13 | 3/3 | 3/3 | 1/1 | 6/6 |
| Q9H4G0-3 | Isoform 3 of Band 4.1-like protein 1 | 0.6±0.2 | 0.00 | 36.7 | 11.4 | 86.5 | 10 | 3/3 | 3/3 | 0/1 | 4/6 |
| P14209-3 | Isoform 3 of CD99 antigen | 0.5±0.1 | 0.00 | 14.6 | 10.7 | 17.1 | 13 | 3/3 | 3/3 | 1/1 | 6/6 |
| P09038-2 | Isoform 3 of Fibroblast growth factor 2 | 0.3±0.1 | 0.00 | 10.1 | 18.7 | 17.2 | 13 | 3/3 | 3/3 | 1/1 | 6/6 |
| P61978-3 | Isoform 3 of Heterogeneous nuclear ribonucleoprotein K | 0.7±0.2 | 0.02 | 514.2 | 66.1 | 48.5 | 8 | 3/3 | 3/3 | 0/1 | 2/6 |
| Q63ZY3-3 | Isoform 3 of KN motif and ankyrin repeat domain-containing protein 2 | 0.6±0.2 | 0.00 | 132.2 | 32.1 | 90.0 | 11 | 2/3 | 3/3 | 1/1 | 5/6 |
| P48059-3 | Isoform 3 of LIM and senescent cell antigen-like-containing domain protein 1 | 0.7±0.1 | 0.00 | 146.0 | 53.2 | 44.4 | 7 | 1/3 | 1/3 | 1/1 | 4/6 |
| P08651-4 | Isoform 3 of Nuclear factor 1 C-type | 0.5±0.1 | 0.00 | 46.5 | 18.7 | 45.4 | 13 | 3/3 | 3/3 | 1/1 | 6/6 |
| Q8IWS0-3 | Isoform 3 of PHD finger protein 6 | 0.7±0.2 | 0.00 | 32.5 | 31.0 | 41.2 | 10 | 3/3 | 2/3 | 1/1 | 4/6 |
| Q8WUF8-3 | Isoform 3 of Protein FAM172A | 0.7±0.1 | 0.00 | 16.0 | 7.0 | 42.8 | 7 | 0/3 | 1/3 | 1/1 | 5/6 |
| Q13813-3 | Isoform 3 of Spectrin alpha chain, non-erythrocytic 1 | 0.6±0.2 | 0.00 | 1852.3 | 73.9 | 282.1 | 11 | 1/3 | 3/3 | 1/1 | 6/6 |
| Q9HA65-3 | Isoform 3 of TBC1 domain family member 17 | 0.7±0.2 | 0.02 | 20.7 | 11.5 | 67.2 | 10 | 2/3 | 2/3 | 1/1 | 5/6 |
| O00159-3 | Isoform 3 of Unconventional myosin-Ic | 0.7±0.2 | 0.00 | 392.3 | 51.5 | 119.6 | 10 | 1/3 | 3/3 | 1/1 | 5/6 |
| O43491-4 | Isoform 4 of Band 4.1-like protein 2 | 0.5±0.1 | 0.00 | 278.6 | 53.4 | 95.5 | 12 | 3/3 | 3/3 | 0/1 | 6/6 |
| P50570-4 | Isoform 4 of Dynamin-2 | 0.6±0.1 | 0.00 | 218.5 | 45.4 | 97.9 | 11 | 2/3 | 3/3 | 1/1 | 5/6 |
| Q15555-4 | Isoform 4 of Microtubule-associated protein RP/EB family member 2 | 0.7±0.2 | 0.02 | 44.9 | 43.8 | 30.7 | 9 | 2/3 | 1/3 | 1/1 | 5/6 |
| P55201-4 | Isoform 4 of Peregrin | 0.3±0.4 | 0.00 | 6.0 | 1.8 | 127.3 | 12 | 3/3 | 3/3 | 1/1 | 5/6 |
| Q9NUN5-4 | Isoform 4 of Probable lysosomal cobalamin transporter | 0.7±0.1 | 0.00 | 9.0 | 9.6 | 21.4 | 7 | 1/3 | 1/3 | 1/1 | 4/6 |
| Q9UN36-4 | Isoform 4 of Protein NDRG2 | 0.4±0.1 | 0.00 | 39.7 | 29.9 | 36.0 | 13 | 3/3 | 3/3 | 1/1 | 6/6 |
| Q9UBP9-4 | Isoform 4 of PTB domain-containing engulfment adapter protein 1 | 0.6±0.1 | 0.00 | 11.7 | 19.2 | 33.3 | 10 | 2/3 | 2/3 | 1/1 | 5/6 |
| P54289-4 | Isoform 4 of Voltage-dependent calcium channel subunit alpha-2/delta-1 | 0.4±0.1 | 0.00 | 116.6 | 23.1 | 121.8 | 13 | 3/3 | 3/3 | 1/1 | 6/6 |
| Q9NY47-4 | Isoform 4 of Voltage-dependent calcium channel subunit alpha-2/delta-2 | 0.4±0.2 | 0.00 | 7.3 | 4.5 | 122.0 | 13 | 3/3 | 3/3 | 1/1 | 6/6 |
| O95319-5 | Isoform 5 of CUGBP Elav-like family member 2 | 0.6±0.1 | 0.00 | 29.5 | 17.6 | 52.0 | 10 | 2/3 | 2/3 | 1/1 | 5/6 |
| Q8N4C8-5 | Isoform 5 of Misshapen-like kinase 1 | 0.7±0.2 | 0.02 | 49.3 | 11.1 | 143.5 | 12 | 3/3 | 2/3 | 1/1 | 6/6 |
| O94901-5 | Isoform 5 of SUN domain-containing protein 1 | 0.6±0.1 | 0.00 | 65.2 | 21.9 | 77.9 | 13 | 3/3 | 3/3 | 1/1 | 6/6 |
| O14639-6 | Isoform 6 of Actin-binding LIM protein 1 | 0.5±0.1 | 0.00 | 89.8 | 31.6 | 84.5 | 13 | 3/3 | 3/3 | 1/1 | 6/6 |
| Q63HR2-6 | Isoform 6 of Tensin-like C1 domain-containing phosphatase | 0.6±0.2 | 0.00 | 45.0 | 10.5 | 152.4 | 10 | 2/3 | 2/3 | 1/1 | 5/6 |
| O94875-7 | Isoform 7 of Sorbin and SH3 domain-containing protein 2 | 0.6±0.3 | 0.01 | 130.3 | 23.9 | 113.5 | 10 | 2/3 | 2/3 | 1/1 | 5/6 |
| Q9UPQ0-9 | Isoform 9 of LIM and calponin homology domains-containing protein 1 | 0.6±0.1 | 0.00 | 9.8 | 4.5 | 100.7 | 11 | 3/3 | 2/3 | 1/1 | 5/6 |
| P36969-2 | Isoform Cytoplasmic of Phospholipid hydroperoxide glutathione peroxidase, mitochondrial | 0.6±0.1 | 0.00 | 52.9 | 62.4 | 19.5 | 13 | 3/3 | 3/3 | 1/1 | 6/6 |
| Q93062-4 | Isoform D of RNA-binding protein with multiple splicing | 0.7±0.2 | 0.03 | 6.2 | 12.6 | 15.8 | 8 | 0/3 | 2/3 | 1/1 | 5/6 |
| Q13557-8 | Isoform Delta 6 of Calcium/calmodulin-dependent protein kinase type II subunit delta | 0.7±0.1 | 0.00 | 139.0 | 45.2 | 54.1 | 7 | 1/3 | 1/3 | 1/1 | 4/6 |
| P42167-2 | Isoform Gamma of Lamina-associated polypeptide 2, isoforms beta/gamma | 0.6±0.2 | 0.00 | 235.4 | 55.7 | 38.7 | 10 | 3/3 | 2/3 | 0/1 | 5/6 |
| P35080-2 | Isoform IIb of Profilin-2 | 0.6±0.2 | 0.00 | 90.5 | 70.7 | 15.1 | 9 | 2/3 | 2/3 | 1/1 | 5/6 |
| P14618-2 | Isoform M1 of Pyruvate kinase PKM | 0.5±0.2 | 0.00 | 1863.3 | 78.2 | 58.0 | 10 | 3/3 | 2/3 | 1/1 | 4/6 |
| P51531-2 | Isoform Short of Probable global transcription activator SNF2L2 | 0.5±0.1 | 0.00 | 96.0 | 14.5 | 179.2 | 13 | 3/3 | 3/3 | 1/1 | 6/6 |
| O95425-4 | Isoform SV4 of Supervillin | 0.6±0.2 | 0.00 | 132.0 | 18.2 | 244.4 | 11 | 2/3 | 3/3 | 1/1 | 5/6 |
| Q7Z4H8 | KDEL motif-containing protein 2 | 0.7±0.1 | 0.00 | 84.1 | 30.0 | 58.5 | 10 | 1/3 | 3/3 | 1/1 | 5/6 |
| Q9HA64 | Ketosamine-3-kinase | 0.6±0.1 | 0.00 | 29.7 | 26.2 | 34.4 | 12 | 3/3 | 2/3 | 1/1 | 6/6 |
| Q96IY1 | Kinetochore-associated protein NSL1 homolog | 0.7±0.1 | 0.00 | 13.4 | 14.2 | 32.1 | 8 | 1/3 | 2/3 | 0/1 | 5/6 |
| J9JID7 | Lamin B2, isoform CRA_a | 0.4±0.1 | 0.00 | 689.4 | 71.1 | 69.9 | 13 | 3/3 | 3/3 | 1/1 | 6/6 |
| Q8N1G4 | Leucine-rich repeat-containing protein 47 | 0.7±0.1 | 0.00 | 174.9 | 51.6 | 63.4 | 10 | 3/3 | 3/3 | 0/1 | 4/6 |
| Q9NZU5 | LIM and cysteine-rich domains protein 1 | 0.6±0.3 | 0.03 | 107.1 | 53.4 | 40.8 | 9 | 2/3 | 1/3 | 1/1 | 5/6 |
| F5H5G1 | Limbic system-associated membrane protein | 0.5±0.3 | 0.00 | 6.4 | 7.3 | 31.7 | 11 | 1/3 | 3/3 | 1/1 | 6/6 |
| Q8IVB5 | LIX1-like protein | 0.5±0.1 | 0.00 | 11.1 | 6.5 | 36.5 | 13 | 3/3 | 3/3 | 1/1 | 6/6 |
| P28330 | Long-chain specific acyl-CoA dehydrogenase, mitochondrial | 0.5±0.2 | 0.01 | 5.8 | 4.4 | 47.6 | 8 | 2/3 | 3/3 | 1/1 | 2/6 |
| Q9NZW5 | MAGUK p55 subfamily member 6 | 0.6±0.1 | 0.00 | 55.0 | 34.6 | 61.1 | 11 | 3/3 | 2/3 | 1/1 | 5/6 |
| P49006 | MARCKS-related protein | 0.6±0.3 | 0.03 | 53.3 | 48.2 | 19.5 | 10 | 2/3 | 2/3 | 0/1 | 6/6 |
| P35625 | Metalloproteinase inhibitor 3 | 0.4±0.4 | 0.02 | 29.2 | 24.6 | 24.1 | 11 | 3/3 | 3/3 | 1/1 | 4/6 |
| A0A0A6YYK0 | Methionine synthase | 0.7±0.1 | 0.00 | 25.6 | 7.9 | 134.7 | 8 | 2/3 | 1/3 | 1/1 | 5/6 |
| P16455 | Methylated-DNA--protein-cysteine methyltransferase | 0.6±0.1 | 0.00 | 62.0 | 47.8 | 21.6 | 12 | 3/3 | 3/3 | 1/1 | 5/6 |
| P51608 | Methyl-CpG-binding protein 2 | 0.5±0.1 | 0.00 | 98.9 | 35.8 | 52.4 | 13 | 3/3 | 3/3 | 1/1 | 6/6 |
| Q02252 | Methylmalonate-semialdehyde dehydrogenase [acylating], mitochondrial | 0.6±0.2 | 0.00 | 313.7 | 59.4 | 57.8 | 11 | 2/3 | 3/3 | 1/1 | 5/6 |
| H0YI09 | Methyltransferase-like protein 7A (Fragment) | 0.6±0.2 | 0.00 | 64.4 | 26.6 | 28.3 | 10 | 2/3 | 3/3 | 1/1 | 4/6 |
| P55083 | Microfibril-associated glycoprotein 4 | 0.2±0.3 | 0.00 | 100.3 | 24.7 | 28.6 | 12 | 3/3 | 3/3 | 1/1 | 5/6 |
| P20774 | Mimecan | 0.5±0.4 | 0.03 | 385.6 | 51.0 | 33.9 | 11 | 1/3 | 3/3 | 1/1 | 6/6 |
| P27361 | Mitogen-activated protein kinase 3 | 0.7±0.1 | 0.00 | 99.3 | 39.1 | 43.1 | 9 | 3/3 | 2/3 | 0/1 | 5/6 |
| Q9H7C9 | Mth938 domain-containing protein | 0.6±0.2 | 0.01 | 26.6 | 58.2 | 13.3 | 11 | 2/3 | 3/3 | 1/1 | 5/6 |
| O95205 | Muscleblind-like 2 (Drosophila), isoform CRA_b | 0.6±0.1 | 0.00 | 38.0 | 25.9 | 28.1 | 11 | 3/3 | 3/3 | 1/1 | 4/6 |
| O95865 | N(G),N(G)-dimethylarginine dimethylaminohydrolase 2 | 0.4±0.1 | 0.00 | 216.9 | 74.7 | 29.6 | 13 | 3/3 | 3/3 | 1/1 | 6/6 |
| Q8NFW8 | N-acylneuraminate cytidylyltransferase | 0.7±0.1 | 0.00 | 116.5 | 51.4 | 48.3 | 10 | 3/3 | 3/3 | 1/1 | 3/6 |
| Q6BCY4 | NADH-cytochrome b5 reductase 2 | 0.6±0.1 | 0.00 | 30.0 | 48.9 | 31.4 | 11 | 3/3 | 2/3 | 0/1 | 6/6 |
| Q8NF91 | Nesprin-1 | 0.5±0.1 | 0.00 | 76.1 | 3.2 | 1010.5 | 12 | 2/3 | 3/3 | 1/1 | 6/6 |
| Q9UMX5 | Neudesin | 0.7±0.1 | 0.00 | 51.3 | 54.7 | 18.8 | 8 | 2/3 | 1/3 | 0/1 | 5/6 |
| A0A087WWD4 | Neural cell adhesion molecule 1 | 0.5±0.2 | 0.00 | 58.6 | 21.4 | 97.3 | 11 | 2/3 | 3/3 | 1/1 | 5/6 |
| Q09666 | Neuroblast differentiation-associated protein AHNAK | 0.7±0.2 | 0.00 | 3818.8 | 83.6 | 628.7 | 9 | 2/3 | 3/3 | 1/1 | 3/6 |
| P05114 | Non-histone chromosomal protein HMG-14 | 0.5±0.2 | 0.00 | 68.2 | 52.0 | 10.7 | 12 | 3/3 | 3/3 | 1/1 | 5/6 |
| P05204 | Non-histone chromosomal protein HMG-17 | 0.4±0.2 | 0.00 | 134.1 | 74.4 | 9.4 | 12 | 3/3 | 3/3 | 1/1 | 5/6 |
| Q8IVI9 | Nostrin | 0.7±0.2 | 0.01 | 6.2 | 8.7 | 57.6 | 7 | 1/3 | 2/3 | 1/1 | 3/6 |
| B1AKN7 | Nuclear factor 1 | 0.5±0.2 | 0.00 | 22.3 | 20.1 | 44.6 | 12 | 3/3 | 2/3 | 0/1 | 6/6 |
| C9JWJ8 | Nuclear factor 1 A-type | 0.6±0.2 | 0.00 | 26.6 | 14.6 | 51.4 | 11 | 3/3 | 2/3 | 1/1 | 6/6 |
| Q14980 | Nuclear mitotic apparatus protein 1 | 0.6±0.2 | 0.00 | 1151.6 | 65.2 | 238.1 | 12 | 3/3 | 3/3 | 1/1 | 5/6 |
| Q9H1E3 | Nuclear ubiquitous casein and cyclin-dependent kinase substrate 1 | 0.5±0.2 | 0.00 | 81.5 | 36.2 | 27.3 | 12 | 3/3 | 3/3 | 0/1 | 6/6 |
| Q9BQ69 | O-acetyl-ADP-ribose deacetylase MACROD1 | 0.7±0.1 | 0.00 | 48.0 | 24.3 | 35.5 | 12 | 3/3 | 3/3 | 1/1 | 5/6 |
| Q6UWY5 | Olfactomedin-like protein 1 | 0.3±0.2 | 0.00 | 176.1 | 52.2 | 45.9 | 12 | 3/3 | 2/3 | 1/1 | 6/6 |
| Q9NRN5 | Olfactomedin-like protein 3 | 0.4±0.2 | 0.00 | 247.9 | 49.8 | 46.0 | 12 | 3/3 | 2/3 | 1/1 | 6/6 |
| F5GX07 | Oligoribonuclease, mitochondrial | 0.7±0.1 | 0.00 | 48.1 | 44.9 | 17.3 | 7 | 1/3 | 2/3 | 1/1 | 3/6 |
| Q96CV9 | Optineurin | 0.6±0.1 | 0.00 | 77.5 | 33.6 | 65.9 | 12 | 3/3 | 3/3 | 1/1 | 5/6 |
| O75781 | Paralemmin-1 | 0.5±0.2 | 0.00 | 31.9 | 20.2 | 42.1 | 12 | 2/3 | 3/3 | 1/1 | 6/6 |
| P20962 | Parathymosin | 0.6±0.2 | 0.00 | 92.6 | 26.5 | 11.5 | 11 | 3/3 | 3/3 | 0/1 | 5/6 |
| O75475 | PC4 and SFRS1-interacting protein | 0.6±0.3 | 0.02 | 230.7 | 50.8 | 60.1 | 10 | 3/3 | 3/3 | 1/1 | 3/6 |
| Q8IYS1 | Peptidase M20 domain-containing protein 2 | 0.7±0.1 | 0.00 | 47.4 | 25.5 | 47.7 | 8 | 2/3 | 2/3 | 1/1 | 3/6 |
| Q13451 | Peptidyl-prolyl cis-trans isomerase FKBP5 | 0.6±0.1 | 0.00 | 119.0 | 51.9 | 51.2 | 12 | 2/3 | 3/3 | 1/1 | 6/6 |
| Q9H2H8 | Peptidyl-prolyl cis-trans isomerase-like 3 | 0.7±0.1 | 0.00 | 28.4 | 37.9 | 18.1 | 10 | 2/3 | 3/3 | 1/1 | 4/6 |
| O60664 | Perilipin-3 | 0.7±0.2 | 0.01 | 224.3 | 69.1 | 47.0 | 8 | 2/3 | 1/3 | 0/1 | 5/6 |
| B4DKF8 | PH and SEC7 domain-containing protein 3 | 0.7±0.2 | 0.00 | 5.8 | 4.8 | 42.4 | 9 | 2/3 | 2/3 | 1/1 | 4/6 |
| P30086 | Phosphatidylethanolamine-binding protein 1 | 0.5±0.1 | 0.00 | 333.7 | 85.6 | 21.0 | 13 | 3/3 | 3/3 | 1/1 | 6/6 |
| P78356 | Phosphatidylinositol 5-phosphate 4-kinase type-2 beta | 0.6±0.1 | 0.00 | 25.7 | 15.9 | 47.3 | 10 | 3/3 | 1/3 | 1/1 | 5/6 |
| P36871 | Phosphoglucomutase-1 | 0.7±0.3 | 0.04 | 241.8 | 68.5 | 61.4 | 10 | 2/3 | 1/3 | 1/1 | 6/6 |
| P18669 | Phosphoglycerate mutase 1 | 0.7±0.1 | 0.00 | 256.0 | 69.7 | 28.8 | 11 | 3/3 | 1/3 | 1/1 | 6/6 |
| Q9H008 | Phospholysine phosphohistidine inorganic pyrophosphate phosphatase | 0.7±0.2 | 0.00 | 17.7 | 22.2 | 29.1 | 7 | 2/3 | 0/3 | 1/1 | 4/6 |
| O60256 | Phosphoribosyl pyrophosphate synthase-associated protein 2 | 0.7±0.1 | 0.00 | 44.4 | 30.9 | 40.9 | 8 | 2/3 | 1/3 | 0/1 | 6/6 |
| Q9H4Z3 | Phosphorylated CTD-interacting factor 1 | 0.7±0.1 | 0.00 | 20.4 | 10.4 | 80.6 | 7 | 3/3 | 1/3 | 0/1 | 3/6 |
| Q5SRE7 | Phytanoyl-CoA dioxygenase domain-containing protein 1 | 0.7±0.1 | 0.00 | 29.0 | 33.3 | 32.4 | 8 | 2/3 | 1/3 | 1/1 | 4/6 |
| P68402 | Platelet-activating factor acetylhydrolase IB subunit beta | 0.7±0.1 | 0.00 | 50.4 | 28.8 | 25.6 | 7 | 3/3 | 1/3 | 1/1 | 2/6 |
| P16234 | Platelet-derived growth factor receptor alpha | 0.5±0.2 | 0.00 | 13.0 | 4.5 | 122.6 | 12 | 3/3 | 3/3 | 1/1 | 5/6 |
| P09619 | Platelet-derived growth factor receptor beta | 0.7±0.2 | 0.00 | 60.6 | 13.5 | 123.9 | 9 | 0/3 | 2/3 | 1/1 | 6/6 |
| Q7Z5L7 | Podocan | 0.6±0.2 | 0.01 | 38.6 | 21.4 | 68.9 | 10 | 1/3 | 3/3 | 1/1 | 5/6 |
| Q6NZI2 | Polymerase I and transcript release factor | 0.4±0.1 | 0.00 | 216.8 | 36.2 | 43.4 | 13 | 3/3 | 3/3 | 1/1 | 6/6 |
| P02545 | Prelamin-A/C | 0.5±0.2 | 0.00 | 1899.7 | 78.8 | 74.1 | 12 | 2/3 | 3/3 | 1/1 | 6/6 |
| Q9BWN1 | Proline-rich protein 14 | 0.7±0.1 | 0.00 | 10.6 | 6.3 | 64.3 | 9 | 3/3 | 2/3 | 1/1 | 3/6 |
| Q9UL18 | Protein argonaute-1 | 0.7±0.2 | 0.01 | 79.1 | 25.4 | 97.2 | 8 | 1/3 | 1/3 | 1/1 | 5/6 |
| Q9H9G7 | Protein argonaute-3 | 0.6±0.1 | 0.00 | 60.5 | 16.4 | 97.3 | 13 | 3/3 | 3/3 | 1/1 | 6/6 |
| Q8N129 | Protein canopy homolog 4 | 0.7±0.2 | 0.01 | 21.1 | 31.1 | 28.3 | 8 | 1/3 | 2/3 | 1/1 | 4/6 |
| Q99497 | Protein DJ-1 | 0.7±0 | 0.00 | 346.4 | 89.4 | 19.9 | 13 | 3/3 | 3/3 | 1/1 | 6/6 |
| P17252 | Protein kinase C alpha type | 0.5±0.1 | 0.00 | 72.8 | 23.8 | 76.7 | 12 | 2/3 | 3/3 | 1/1 | 6/6 |
| Q9UKS6 | Protein kinase C and casein kinase substrate in neurons protein 3 | 0.7±0.1 | 0.00 | 40.8 | 24.3 | 48.5 | 10 | 3/3 | 2/3 | 1/1 | 4/6 |
| Q969G5 | Protein kinase C delta-binding protein | 0.4±0.2 | 0.00 | 43.1 | 31.8 | 27.7 | 12 | 3/3 | 3/3 | 1/1 | 5/6 |
| P60903 | Protein S100-A10 | 0.5±0.2 | 0.00 | 81.6 | 49.5 | 11.2 | 12 | 2/3 | 3/3 | 1/1 | 6/6 |
| A6NIH7 | Protein unc-119 homolog B | 0.7±0.2 | 0.01 | 20.0 | 25.5 | 28.1 | 8 | 3/3 | 1/3 | 0/1 | 4/6 |
| Q5TEH8 | Protein Wnt | 0.3±0.2 | 0.00 | 5.9 | 4.4 | 33.9 | 8 | 2/3 | 3/3 | 1/1 | 2/6 |
| Q5T013 | Putative hydroxypyruvate isomerase | 0.6±0.1 | 0.00 | 21.1 | 30.3 | 30.4 | 13 | 3/3 | 3/3 | 1/1 | 6/6 |
| A0A087WT18 | Putative monooxygenase p33MONOX | 0.6±0.2 | 0.00 | 9.1 | 14.7 | 24.5 | 9 | 3/3 | 3/3 | 1/1 | 2/6 |
| Q9H1K0 | Rabenosyn-5 | 0.7±0.2 | 0.01 | 6.0 | 2.9 | 88.8 | 7 | 1/3 | 2/3 | 0/1 | 4/6 |
| P35241 | Radixin | 0.6±0.1 | 0.00 | 478.3 | 69.1 | 68.5 | 11 | 2/3 | 2/3 | 1/1 | 6/6 |
| Q6IQ22 | Ras-related protein Rab-12 | 0.7±0.2 | 0.01 | 36.4 | 22.1 | 27.2 | 8 | 2/3 | 2/3 | 1/1 | 3/6 |
| Q92930 | Ras-related protein Rab-8B | 0.7±0.1 | 0.00 | 111.8 | 48.3 | 23.6 | 10 | 1/3 | 3/3 | 1/1 | 5/6 |
| P61224 | Ras-related protein Rap-1b | 0.7±0.1 | 0.00 | 174.9 | 71.2 | 20.8 | 10 | 3/3 | 2/3 | 0/1 | 5/6 |
| Q96L35 | Receptor protein-tyrosine kinase | 0.6±0.2 | 0.00 | 11.5 | 4.1 | 102.5 | 11 | 2/3 | 3/3 | 0/1 | 6/6 |
| Q15493 | Regucalcin | 0.3±0.1 | 0.00 | 5.7 | 11.0 | 33.2 | 13 | 3/3 | 3/3 | 1/1 | 6/6 |
| Q9P2K3 | REST corepressor 3 | 0.7±0.2 | 0.02 | 39.5 | 16.2 | 55.5 | 8 | 3/3 | 2/3 | 0/1 | 3/6 |
| P00352 | Retinal dehydrogenase 1 | 0.4±0.2 | 0.00 | 501.4 | 60.9 | 54.8 | 12 | 2/3 | 3/3 | 1/1 | 6/6 |
| P09455 | Retinol-binding protein 1 | 0.3±0.1 | 0.00 | 218.8 | 78.5 | 15.8 | 13 | 3/3 | 3/3 | 1/1 | 6/6 |
| O95980 | Reversion-inducing cysteine-rich protein with Kazal motifs | 0.6±0.2 | 0.00 | 14.5 | 6.8 | 106.4 | 11 | 2/3 | 3/3 | 1/1 | 5/6 |
| A1A4S6 | Rho GTPase-activating protein 10 | 0.7±0.2 | 0.03 | 7.9 | 2.9 | 89.3 | 8 | 0/3 | 2/3 | 1/1 | 5/6 |
| P13489 | Ribonuclease inhibitor | 0.7±0.1 | 0.00 | 270.2 | 77.4 | 49.9 | 8 | 1/3 | 1/3 | 1/1 | 5/6 |
| P60891 | Ribose-phosphate pyrophosphokinase 1 | 0.6±0.1 | 0.00 | 124.7 | 50.0 | 34.8 | 9 | 2/3 | 2/3 | 0/1 | 5/6 |
| Q5TD07 | Ribosyldihydronicotinamide dehydrogenase [quinone] | 0.5±0.2 | 0.00 | 17.9 | 38.9 | 21.5 | 13 | 3/3 | 3/3 | 1/1 | 6/6 |
| Q5EBL4 | RILP-like protein 1 | 0.6±0.2 | 0.00 | 7.0 | 4.5 | 47.1 | 10 | 3/3 | 2/3 | 0/1 | 5/6 |
| Q9C0B0 | RING finger protein unkempt homolog | 0.7±0.1 | 0.00 | 13.5 | 4.7 | 88.0 | 8 | 1/3 | 3/3 | 0/1 | 4/6 |
| Q96E39 | RNA binding motif protein, X-linked-like-1 | 0.6±0.2 | 0.00 | 184.8 | 36.9 | 42.1 | 11 | 3/3 | 3/3 | 1/1 | 4/6 |
| Q5TZA2 | Rootletin | 0.6±0.1 | 0.00 | 107.8 | 15.9 | 228.4 | 13 | 3/3 | 3/3 | 1/1 | 6/6 |
| Q12765 | Secernin-1 | 0.7±0.2 | 0.01 | 48.3 | 20.3 | 46.4 | 7 | 2/3 | 2/3 | 1/1 | 2/6 |
| Q96FV2 | Secernin-2 | 0.6±0.1 | 0.00 | 8.9 | 3.1 | 46.6 | 11 | 3/3 | 2/3 | 1/1 | 5/6 |
| Q13228 | Selenium-binding protein 1 | 0.7±0.2 | 0.02 | 312.8 | 69.5 | 52.4 | 8 | 2/3 | 3/3 | 0/1 | 4/6 |
| A0A087WYP2 | Selenoprotein H | 0.7±0.3 | 0.04 | 70.5 | 44.6 | 13.3 | 9 | 3/3 | 3/3 | 1/1 | 2/6 |
| Q8WYJ6 | Septin-1 | 0.7±0.2 | 0.01 | 9.0 | 10.4 | 41.9 | 7 | 1/3 | 2/3 | 1/1 | 3/6 |
| E7EW69 | Septin-10 | 0.5±0.1 | 0.00 | 78.5 | 48.0 | 52.1 | 13 | 3/3 | 3/3 | 1/1 | 6/6 |
| Q15019 | Septin-2 | 0.7±0.2 | 0.01 | 317.3 | 74.2 | 41.5 | 7 | 1/3 | 1/3 | 1/1 | 4/6 |
| P35237 | Serpin B6 | 0.5±0.2 | 0.00 | 202.6 | 70.0 | 42.6 | 12 | 3/3 | 3/3 | 1/1 | 5/6 |
| P02743 | Serum amyloid P-component | 0.2±0.1 | 0.00 | 159.2 | 33.6 | 25.4 | 13 | 3/3 | 3/3 | 1/1 | 6/6 |
| O95810 | Serum deprivation-response protein | 0.5±0.1 | 0.00 | 66.4 | 18.4 | 47.1 | 13 | 3/3 | 3/3 | 1/1 | 6/6 |
| P10768 | S-formylglutathione hydrolase | 0.6±0.1 | 0.00 | 161.9 | 50.7 | 31.4 | 13 | 3/3 | 3/3 | 1/1 | 6/6 |
| P51692 | Signal transducer and activator of transcription 5B | 0.6±0.2 | 0.00 | 79.3 | 23.0 | 89.8 | 9 | 2/3 | 2/3 | 1/1 | 4/6 |
| P42226 | Signal transducer and activator of transcription 6 | 0.7±0.1 | 0.00 | 62.1 | 22.8 | 94.1 | 8 | 1/3 | 2/3 | 0/1 | 5/6 |
| Q53HV7 | Single-strand selective monofunctional uracil DNA glycosylase | 0.5±0.1 | 0.00 | 6.9 | 8.2 | 29.8 | 8 | 1/3 | 1/3 | 1/1 | 5/6 |
| Q13126 | S-methyl-5'-thioadenosine phosphorylase | 0.7±0.1 | 0.00 | 144.1 | 78.1 | 31.2 | 7 | 2/3 | 2/3 | 1/1 | 2/6 |
| C9J0K6 | Sorcin | 0.7±0.1 | 0.00 | 120.8 | 90.3 | 17.6 | 10 | 1/3 | 2/3 | 1/1 | 6/6 |
| A0A087WUZ3 | Spectrin beta chain, non-erythrocytic 1 | 0.6±0.1 | 0.00 | 1449.1 | 69.6 | 274.7 | 12 | 2/3 | 3/3 | 1/1 | 6/6 |
| P52788 | Spermine synthase | 0.7±0.1 | 0.00 | 79.9 | 48.9 | 41.2 | 11 | 2/3 | 2/3 | 1/1 | 6/6 |
| Q9Y657 | Spindlin-1 | 0.5±0.1 | 0.00 | 20.0 | 30.9 | 29.6 | 13 | 3/3 | 3/3 | 1/1 | 6/6 |
| P16949 | Stathmin | 0.6±0.3 | 0.04 | 172.7 | 83.9 | 17.3 | 10 | 3/3 | 3/3 | 0/1 | 4/6 |
| Q14683 | Structural maintenance of chromosomes protein 1A | 0.7±0.2 | 0.00 | 467.4 | 65.0 | 143.1 | 8 | 2/3 | 3/3 | 1/1 | 2/6 |
| Q9UQE7 | Structural maintenance of chromosomes protein 3 | 0.7±0.1 | 0.00 | 433.1 | 57.8 | 141.5 | 8 | 1/3 | 3/3 | 1/1 | 3/6 |
| Q9UH99 | SUN domain-containing protein 2 | 0.7±0.1 | 0.00 | 167.5 | 35.7 | 80.3 | 10 | 1/3 | 3/3 | 1/1 | 5/6 |
| P00441 | Superoxide dismutase [Cu-Zn] | 0.6±0.2 | 0.01 | 199.0 | 90.9 | 15.9 | 10 | 3/3 | 2/3 | 0/1 | 5/6 |
| Q99536 | Synaptic vesicle membrane protein VAT-1 homolog | 0.5±0.1 | 0.00 | 180.0 | 43.3 | 41.9 | 13 | 3/3 | 3/3 | 1/1 | 6/6 |
| P61764 | Syntaxin-binding protein 1 | 0.6±0.1 | 0.00 | 26.4 | 14.1 | 67.5 | 12 | 3/3 | 3/3 | 1/1 | 5/6 |
| Q9Y4G6 | Talin-2 | 0.7±0.1 | 0.00 | 570.9 | 43.5 | 271.4 | 10 | 2/3 | 1/3 | 1/1 | 6/6 |
| Q13148 | TAR DNA-binding protein 43 | 0.7±0.2 | 0.01 | 93.8 | 45.7 | 44.7 | 8 | 3/3 | 3/3 | 0/1 | 2/6 |
| Q7Z7G0 | Target of Nesh-SH3 | 0.5±0.2 | 0.00 | 36.4 | 9.4 | 118.6 | 11 | 3/3 | 2/3 | 1/1 | 5/6 |
| Q68CZ2 | Tensin-3 | 0.7±0.1 | 0.00 | 107.7 | 18.2 | 155.2 | 10 | 2/3 | 2/3 | 1/1 | 5/6 |
| Q9P016 | Thymocyte nuclear protein 1 | 0.7±0.2 | 0.01 | 65.1 | 55.1 | 25.7 | 9 | 3/3 | 3/3 | 1/1 | 2/6 |
| P37837 | Transaldolase | 0.5±0.1 | 0.00 | 280.5 | 54.3 | 37.5 | 12 | 3/3 | 2/3 | 1/1 | 6/6 |
| Q969E4 | Transcription elongation factor A protein-like 3 | 0.4±0.2 | 0.00 | 30.5 | 36.0 | 22.5 | 13 | 3/3 | 3/3 | 1/1 | 6/6 |
| Q00577 | Transcriptional activator protein Pur-alpha | 0.6±0.1 | 0.00 | 89.1 | 43.2 | 34.9 | 12 | 2/3 | 3/3 | 1/1 | 6/6 |
| Q96EM0 | Trans-L-3-hydroxyproline dehydratase | 0.7±0.2 | 0.04 | 20.6 | 20.9 | 38.1 | 7 | 1/3 | 1/3 | 1/1 | 4/6 |
| Q9Y2S6 | Translation machinery-associated protein 7 | 0.7±0.2 | 0.01 | 44.5 | 65.6 | 7.1 | 10 | 3/3 | 3/3 | 0/1 | 4/6 |
| Q9BTV4 | Transmembrane protein 43 | 0.6±0.1 | 0.00 | 130.0 | 42.3 | 44.8 | 11 | 2/3 | 3/3 | 1/1 | 5/6 |
| P28289 | Tropomodulin-1 | 0.5±0.2 | 0.00 | 94.0 | 60.2 | 40.5 | 11 | 3/3 | 3/3 | 1/1 | 4/6 |
| Q9NZR1 | Tropomodulin-2 | 0.6±0.1 | 0.00 | 13.1 | 8.8 | 39.6 | 13 | 3/3 | 3/3 | 1/1 | 6/6 |
| Q9GZM7 | Tubulointerstitial nephritis antigen-like | 0.6±0.2 | 0.00 | 144.1 | 45.8 | 52.4 | 11 | 3/3 | 2/3 | 1/1 | 5/6 |
| P09936 | Ubiquitin carboxyl-terminal hydrolase isozyme L1 | 0.4±0.1 | 0.00 | 109.2 | 65.9 | 24.8 | 13 | 3/3 | 3/3 | 1/1 | 6/6 |
| Q9BZV1 | UBX domain-containing protein 6 | 0.6±0.1 | 0.00 | 86.6 | 48.8 | 49.7 | 11 | 2/3 | 3/3 | 1/1 | 5/6 |
| Q96AT1 | Uncharacterized protein KIAA1143 | 0.7±0.2 | 0.04 | 7.4 | 12.3 | 17.5 | 10 | 3/3 | 3/3 | 0/1 | 4/6 |
| Q9NUL5 | UPF0515 protein C19orf66 | 0.7±0.2 | 0.05 | 25.4 | 23.7 | 33.1 | 9 | 2/3 | 1/3 | 1/1 | 5/6 |
| Q5T6V5 | UPF0553 protein C9orf64 | 0.6±0.1 | 0.00 | 7.9 | 6.5 | 39.0 | 13 | 3/3 | 3/3 | 1/1 | 6/6 |
| Q9GZN8 | UPF0687 protein C20orf27 | 0.6±0.1 | 0.00 | 10.9 | 8.1 | 19.3 | 12 | 3/3 | 2/3 | 1/1 | 6/6 |
| Q9H3H3 | UPF0696 protein C11orf68 | 0.7±0.1 | 0.00 | 18.5 | 20.3 | 27.3 | 8 | 2/3 | 2/3 | 1/1 | 3/6 |
| P46939 | Utrophin | 0.7±0.1 | 0.00 | 488.5 | 34.5 | 394.2 | 10 | 2/3 | 2/3 | 1/1 | 5/6 |
| Q9P035 | Very-long-chain (3R)-3-hydroxyacyl-CoA dehydratase 3 | 0.7±0.2 | 0.01 | 48.3 | 21.6 | 43.1 | 7 | 1/3 | 2/3 | 1/1 | 3/6 |
| P08670 | Vimentin | 0.7±0.1 | 0.00 | 4918.5 | 93.1 | 53.6 | 7 | 1/3 | 2/3 | 0/1 | 4/6 |
| O60504 | Vinexin | 0.6±0.1 | 0.00 | 65.6 | 26.2 | 75.3 | 10 | 2/3 | 2/3 | 1/1 | 5/6 |
| F8VUW8 | Voltage-dependent L-type calcium channel subunit beta-3 (Fragment) | 0.4±0.1 | 0.00 | 5.0 | 4.9 | 13.5 | 8 | 2/3 | 3/3 | 1/1 | 2/6 |
| A0A0A0MSG0 | WAS/WASL-interacting protein family member 3 | 0.6±0.2 | 0.00 | 9.5 | 9.5 | 49.3 | 10 | 2/3 | 1/3 | 1/1 | 6/6 |
| O76076 | WNT1-inducible-signaling pathway protein 2 | 0.4±0.2 | 0.00 | 25.6 | 28.0 | 26.8 | 13 | 3/3 | 3/3 | 1/1 | 6/6 |
| O76024 | Wolframin | 0.6±0.2 | 0.00 | 38.4 | 14.5 | 100.2 | 11 | 2/3 | 3/3 | 0/1 | 6/6 |
| P13010 | X-ray repair cross-complementing protein 5 | 0.7±0.2 | 0.03 | 687.8 | 65.9 | 82.7 | 9 | 2/3 | 3/3 | 1/1 | 3/6 |
| P12956 | X-ray repair cross-complementing protein 6 | 0.7±0.2 | 0.02 | 595.3 | 66.2 | 69.8 | 8 | 3/3 | 3/3 | 1/1 | 2/6 |
| Q96GY0 | Zinc finger C2HC domain-containing protein 1A | 0.6±0.2 | 0.00 | 8.1 | 8.3 | 35.1 | 9 | 2/3 | 2/3 | 0/1 | 5/6 |
| Q96NB3 | Zinc finger protein 830 | 0.7±0.1 | 0.01 | 14.7 | 11.0 | 42.0 | 7 | 2/3 | 2/3 | 0/1 | 3/6 |
