# Supplementary material for: Identification and validation of differentially expressed proteins in epithelial ovarian cancers using quantitative proteomics
Source: Oncotarget. 2016 Nov 4;7(50):83187–99. doi: 10.18632/oncotarget.13077 (PMC5347761; doi:10.18632/oncotarget.13077)
Supplement: Supplementary file 4 [file oncotarget-07-83187-s004.docx]

Table S4. Up regulated proteins in A2780 CLIC1 KD cell line compared withA2780 NCi cell line. Ratio 1 and 2 stand for the ratios of two biological repeats of the quantitative proteomics with A2780 CLIC1 KD cells and A2780 NCi cells.

| Accession | Protein Description | Ratio 1 of CLIC1 KD/Nci | Ratio 2 CLIC1 KD/Nci | Score | Cover  age | MW (kDa) |
| --- | --- | --- | --- | --- | --- | --- |
| P31947 | 14-3-3 protein sigma | 1.5 | 1.6 | 160.4 | 56.9 | 27.8 |
| F8VR69 | 60S ribosomal protein L6 (Fragment) | 2.0 | 2.2 | 41.0 | 74.6 | 7.2 |
| P30520 | Adenylosuccinate synthetase isozyme 2 | 1.7 | 1.6 | 305.9 | 59.2 | 50.1 |
| H3BU49 | ADP-ribosylation factor-like protein 2-binding protein | 1.7 | 1.7 | 16.6 | 12.2 | 13.8 |
| Q8WXK1 | Ankyrin repeat and SOCS box protein 15 | 2.0 | 2.5 | 5.1 | 1.4 | 65.8 |
| Q9HBK9 | Arsenite methyltransferase | 1.5 | 1.6 | 41.1 | 27.2 | 41.7 |
| H0YD26 | ATP-dependent RNA helicase DDX25 (Fragment) | 1.6 | 1.5 | 8.1 | 23.6 | 21.0 |
| P50895 | Basal cell adhesion molecule | 1.6 | 1.6 | 18.4 | 11.3 | 67.4 |
| Q8N1Q1 | Carbonic anhydrase 13 | 1.6 | 1.6 | 5.5 | 6.5 | 29.4 |
| Q9ULX7 | Carbonic anhydrase 14 | 1.7 | 1.6 | 12.4 | 13.4 | 37.6 |
| Q5SVL2 | Caspase-7 (Fragment) | 1.7 | 1.6 | 42.1 | 51.8 | 24.8 |
| O95971 | CD160 antigen | 3.4 | 3.8 | 8.2 | 6.1 | 19.8 |
| P00450 | Ceruloplasmin | 1.9 | 1.8 | 100.6 | 22.0 | 122.1 |
| E9PFZ2 | Ceruloplasmin | 1.8 | 1.8 | 99.5 | 24.5 | 108.8 |
| O95833 | Chloride intracellular channel protein 3 | 1.6 | 1.6 | 77.3 | 63.1 | 26.6 |
| Q8IYE1 | Coiled-coil domain-containing protein 13 | 2.6 | 4.0 | 5.2 | 1.7 | 80.8 |
| E7ENY8 | Collagen alpha-1(III) chain | 2.5 | 2.5 | 8.2 | 2.4 | 111.9 |
| P08603 | Complement factor H | 1.6 | 1.6 | 142.8 | 33.6 | 139.0 |
| B4DR67 | Dolichyl-phosphate beta-glucosyltransferase | 1.8 | 1.8 | 16.5 | 27.8 | 25.3 |
| Q9UKM7 | Endoplasmic reticulum mannosyl-oligosaccharide 1,2-alpha-mannosidase | 1.7 | 1.5 | 7.6 | 4.3 | 79.5 |
| Q96HE7 | ERO1-like protein alpha | 1.5 | 1.6 | 70.1 | 28.4 | 54.4 |
| Q16658 | Fascin | 1.5 | 1.6 | 118.8 | 48.7 | 54.5 |
| P30279 | G1/S-specific cyclin-D2 | 1.7 | 1.7 | 7.2 | 11.8 | 33.0 |
| Q08380 | Galectin-3-binding protein | 2.2 | 2.1 | 18.5 | 10.4 | 65.3 |
| B7ZC06 | Golgin subfamily A member 2 (Fragment) | 1.5 | 1.7 | 78.1 | 44.6 | 53.4 |
| Q96D09 | G-protein coupled receptor-associated sorting protein 2 | 1.9 | 1.7 | 9.5 | 5.5 | 93.7 |
| P02751-12 | Isoform 12 of Fibronectin | 1.5 | 1.8 | 9.3 | 2.9 | 221.2 |
| O75891-2 | Isoform 2 of Cytosolic 10-formyltetrahydrofolate dehydrogenase | 5.1 | 5.5 | 6.8 | 1.5 | 87.5 |
| A2RTX5-2 | Isoform 2 of Probable threonine--tRNA ligase 2, cytoplasmic | 2.0 | 2.3 | 32.9 | 4.5 | 81.5 |
| O00204-2 | Isoform 2 of Sulfotransferase family cytosolic 2B member 1 | 2.1 | 2.1 | 84.1 | 36.3 | 39.6 |
| Q7Z7G8-2 | Isoform 2 of Vacuolar protein sorting-associated protein 13B | 2.2 | 2.4 | 5.2 | 0.2 | 445.7 |
| Q02952-3 | Isoform 3 of A-kinase anchor protein 12 | 1.7 | 1.8 | 118.8 | 21.5 | 180.9 |
| Q5J8M3-3 | Isoform 3 of ER membrane protein complex subunit 4 | 1.5 | 1.7 | 13.0 | 36.0 | 15.1 |
| P10909-4 | Isoform 4 of Clusterin | 1.8 | 1.7 | 46.6 | 21.4 | 48.8 |
| P04731 | Metallothionein-1A | 3.1 | 2.7 | 17.6 | 45.9 | 6.1 |
| D6R9P2 | Mitochondrial sodium/hydrogen exchanger 9B2 | 1.8 | 1.7 | 15.0 | 40.2 | 12.4 |
| Q5T2W1 | Na(+)/H(+) exchange regulatory cofactor NHE-RF3 | 3.9 | 3.8 | 674.1 | 69.9 | 57.1 |
| D6RH06 | PDZ and LIM domain protein 7 (Fragment) | 1.9 | 2.2 | 151.9 | 63.4 | 30.5 |
| P45877 | Peptidyl-prolyl cis-trans isomerase C | 1.7 | 1.7 | 37.8 | 24.1 | 22.7 |
| C9J363 | Programmed cell death 10, isoform CRA_b | 2.4 | 2.1 | 33.8 | 55.7 | 17.5 |
| Q9BZQ8 | Protein Niban | 1.6 | 1.7 | 42.6 | 13.3 | 103.1 |
| P26447 | Protein S100-A4 | 3.2 | 3.2 | 33.9 | 49.5 | 11.7 |
| O43609 | Protein sprouty homolog 1 | 2.1 | 1.9 | 39.9 | 22.6 | 35.1 |
| Q13637 | Ras-related protein Rab-32 | 1.6 | 1.9 | 7.2 | 8.4 | 25.0 |
| Q5SYQ7 | Retinal dehydrogenase 1 (Fragment) | 3.9 | 4.1 | 360.4 | 68.5 | 22.6 |
| P00352 | Retinal dehydrogenase 1 | 5.1 | 5.3 | 1100.4 | 77.6 | 54.8 |
| O75911 | Short-chain dehydrogenase/reductase 3 | 2.0 | 1.9 | 23.1 | 23.8 | 33.5 |
| E9PKW4 | Sulfotransferase 1A2 | 1.9 | 2.5 | 7.2 | 12.6 | 29.0 |
| M0QZS6 | SUMO-activating enzyme subunit 1 | 1.7 |  | 121.9 | 64.9 | 29.4 |
| O95049 | Tight junction protein ZO-3 | 1.8 | 1.8 | 25.9 | 15.8 | 101.3 |
| B7WP88 | TPR and ankyrin repeat-containing protein 1 | 1.7 | 1.9 | 6.7 | 0.3 | 273.3 |
| H0YLN8 | Transient receptor potential cation channel subfamily M member 7 | 1.7 | 1.9 | 33.3 | 6.4 | 212.5 |
