# Supplementary material for: Identification and validation of differentially expressed proteins in epithelial ovarian cancers using quantitative proteomics
Source: Oncotarget. 2016 Nov 4;7(50):83187–99. doi: 10.18632/oncotarget.13077 (PMC5347761; doi:10.18632/oncotarget.13077)
Supplement: Supplementary file 5 [file oncotarget-07-83187-s005.docx]

Table S5. Down regulated proteins in A2780 CLIC1 KD cell line compared withA2780 NCi cell line. Ratio 1 and 2 stand for the ratios of two biological repeats of the quantitative proteomics with A2780 CLIC1 KD cells and A2780 NCi cells.

| Accession | Protein Description | Ratio 1 of CLIC1 KD/Nci | Ratio 2 CLIC1 KD/Nci | Score | Cover  age | MW (kDa) |
| --- | --- | --- | --- | --- | --- | --- |
| Q8IV48 | 3'-5' exoribonuclease 1 | 0.7 | 0.7 | 117.1 | 42.7 | 40.0 |
| P62847 | 40S ribosomal protein S24 | 0.7 | 0.6 | 289.8 | 65.4 | 15.4 |
| Q9BPX5 | Actin-related protein 2/3 complex subunit 5-like protein | 0.7 | 0.7 | 62.1 | 58.8 | 16.9 |
| P40617 | ADP-ribosylation factor-like protein 4A | 0.4 | 0.5 | 8.3 | 13.0 | 22.6 |
| E9PHY5 | Band 4.1-like protein 2 | 0.5 | 0.5 | 178.9 | 35.6 | 104.3 |
| F5GXJ9 | CD166 antigen | 0.4 | 0.4 | 25.4 | 13.9 | 59.5 |
| Q7LBR1 | Charged multivesicular body protein 1b | 0.7 | 0.7 | 28.0 | 31.7 | 22.1 |
| O00299 | Chloride intracellular channel protein 1 | 0.7 | 0.6 | 489.8 | 88.4 | 26.9 |
| P29279 | Connective tissue growth factor | 0.4 | 0.4 | 25.6 | 23.5 | 38.1 |
| P17812 | CTP synthase 1 | 0.6 | 0.6 | 656.2 | 66.2 | 66.6 |
| P15924 | Desmoplakin | 0.6 | 0.6 | 468.5 | 40.8 | 331.6 |
| Q16555 | Dihydropyrimidinase-related protein 2 | 0.7 | 0.7 | 292.2 | 68.0 | 62.3 |
| Q96G61 | Diphosphoinositol polyphosphate phosphohydrolase 3-beta | 0.7 | 0.6 | 46.2 | 29.9 | 18.5 |
| Q6PJ61 | F-box only protein 46 | 0.7 | 0.6 | 12.6 | 5.3 | 64.6 |
| P49448 | Glutamate dehydrogenase 2, mitochondrial | 0.7 | 0.6 | 152.3 | 30.5 | 61.4 |
| E7EU81 | Golgin subfamily B member 1 (Fragment) | 0.7 | 0.6 | 171.4 | 27.7 | 188.1 |
| P54652 | Heat shock-related 70 kDa protein 2 | 0.7 | 0.5 | 1051.5 | 25.4 | 70.0 |
| P09601 | Heme oxygenase 1 | 0.7 | 0.7 | 176.9 | 54.5 | 32.8 |
| Q5JSK7 | High mobility group nucleosome-binding domain-containing protein 5 (Fragment) | 0.5 | 0.6 | 7.2 | 16.9 | 9.9 |
| O60814 | Histone H2B type 1-K | 0.7 | 0.5 | 1812.5 | 88.1 | 13.9 |
| Q86YM6 | HOMER1F | 0.5 | 0.6 | 7.2 | 21.7 | 20.8 |
| E7EMF1 | Integrin alpha-2 | 0.7 | 0.6 | 13.9 | 7.2 | 88.5 |
| P18206-2 | Isoform 1 of Vinculin | 0.7 | 0.7 | 3280.0 | 89.7 | 116.6 |
| Q12797-10 | Isoform 10 of Aspartyl/asparaginyl beta-hydroxylase | 0.5 | 0.5 | 181.4 | 39.9 | 83.2 |
| P04035-2 | Isoform 2 of 3-hydroxy-3-methylglutaryl-coenzyme A reductase | 0.7 | 0.6 | 14.1 | 6.4 | 92.0 |
| Q6FIF0-2 | Isoform 2 of AN1-type zinc finger protein 6 | 0.7 | 0.7 | 24.7 | 23.0 | 21.4 |
| Q16828-2 | Isoform 2 of Dual specificity protein phosphatase 6 | 0.6 | 0.6 | 11.2 | 19.6 | 26.5 |
| Q08431-2 | Isoform 2 of Lactadherin | 0.6 | 0.7 | 10.3 | 16.7 | 35.2 |
| Q53GG5-2 | Isoform 2 of PDZ and LIM domain protein 3 | 0.6 | 0.5 | 60.1 | 35.8 | 34.3 |
| Q9BY77-2 | Isoform 2 of Polymerase delta-interacting protein 3 | 0.6 | 0.6 | 116.6 | 57.4 | 42.9 |
| Q9Y2Z0-2 | Isoform 2 of Suppressor of G2 allele of SKP1 homolog | 0.7 | 0.7 | 188.8 | 65.8 | 37.8 |
| O00716-2 | Isoform 2 of Transcription factor E2F3 | 0.7 | 0.7 | 5.4 | 4.5 | 37.0 |
| Q9NZA1-3 | Isoform 3 of Chloride intracellular channel protein 5 | 0.7 | 0.7 | 14.5 | 11.7 | 22.8 |
| Q14677-3 | Isoform 3 of Clathrin interactor 1 | 0.7 | 0.6 | 101.8 | 35.6 | 70.2 |
| Q8WW22-3 | Isoform 3 of DnaJ homolog subfamily A member 4 | 0.7 | 0.7 | 29.5 | 11.4 | 41.6 |
| P06748-3 | Isoform 3 of Nucleophosmin | 0.7 | 0.6 | 1755.2 | 63.3 | 28.4 |
| Q96LZ7-3 | Isoform 3 of Regulator of microtubule dynamics protein 2 | 0.7 | 0.6 | 6.5 | 10.4 | 24.8 |
| Q8IYB5-3 | Isoform 3 of Stromal membrane-associated protein 1 | 0.7 | 0.6 | 71.3 | 28.2 | 47.8 |
| Q05682-4 | Isoform 4 of Caldesmon | 0.6 | 0.7 | 354.2 | 66.9 | 62.6 |
| P04637-4 | Isoform 4 of Cellular tumor antigen p53 | 0.7 | 0.7 | 45.5 | 29.4 | 39.3 |
| Q86UP2-4 | Isoform 4 of Kinectin | 0.6 | 0.5 | 940.9 | 70.3 | 152.9 |
| P35580-4 | Isoform 4 of Myosin-10 | 0.7 | 0.6 | 1173.2 | 60.7 | 232.4 |
| Q9UKE5-8 | Isoform 8 of TRAF2 and NCK-interacting protein kinase | 0.6 | 0.5 | 6.5 | 3.6 | 144.2 |
| P49023-2 | Isoform Alpha of Paxillin | 0.4 | 0.4 | 167.8 | 48.3 | 60.9 |
| Q5H9A7 | Metalloproteinase inhibitor 1 | 0.7 | 0.7 | 48.5 | 71.3 | 16.0 |
| P78316 | Nucleolar protein 14 | 0.7 | 0.7 | 54.7 | 14.2 | 97.6 |
| F5GZ78 | Paxillin | 0.4 | 0.4 | 162.0 | 45.8 | 64.2 |
| P30405 | Peptidyl-prolyl cis-trans isomerase F, mitochondrial | 0.7 | 0.8 | 140.8 | 78.7 | 22.0 |
| Q9HAH7 | Probable fibrosin-1 | 0.7 | 0.5 | 11.1 | 5.4 | 48.4 |
| Q9C004 | Protein sprouty homolog 4 | 0.7 | 0.7 | 33.9 | 22.1 | 32.5 |
| Q99519 | Sialidase-1 | 0.7 | 0.7 | 31.3 | 22.4 | 45.4 |
| F5GYJ5 | Succinate dehydrogenase assembly factor 2, mitochondrial | 0.7 | 0.5 | 10.5 | 9.1 | 18.4 |
| P51687 | Sulfite oxidase, mitochondrial | 0.6 | 0.6 | 10.5 | 7.5 | 60.2 |
| P07951 | Tropomyosin beta chain | 0.7 | 0.7 | 1032.6 | 91.9 | 32.8 |
| P62987 | Ubiquitin-60S ribosomal protein L40 | 0.7 | 0.5 | 327.5 | 75.0 | 14.7 |
| Q92575 | UBX domain-containing protein 4 | 0.7 | 0.7 | 95.3 | 27.2 | 56.7 |
| P30085 | UMP-CMP kinase | 0.7 | 0.7 | 231.5 | 69.4 | 22.2 |
| Q5JVM0 | Unconventional myosin-VI (Fragment) | 0.4 | 0.4 | 63.6 | 40.2 | 20.6 |
| Q16831 | Uridine phosphorylase 1 | 0.7 | 0.7 | 32.6 | 17.4 | 33.9 |
| O95625 | Zinc finger and BTB domain-containing protein 11 | 0.6 | 0.6 | 18.5 | 3.1 | 119.3 |
| Q9H2Y7 | Zinc finger protein 106 | 0.7 | 0.7 | 12.6 | 1.9 | 208.8 |
| E7ENT5 | Zinc transporter ZIP10 (Fragment) | 0.7 | 0.7 | 8.3 | 23.8 | 14.8 |
